# Supplementary material for: Decoupled charge and heat transport in Fe2VAl composite thermoelectrics with topological-insulating grain boundary networks
Source: Nat Commun. 2025 Mar 26;16:2976. doi: 10.1038/s41467-025-57250-6 (PMC11947127; doi:10.1038/s41467-025-57250-6)
Supplement: Supplementary file 1 — Supplementary Information [file 41467_2025_57250_MOESM1_ESM.pdf]

Supplementary Information for

Decoupled charge and heat transport in Fe_2VAl composite thermoelectrics with topological-insulating grain boundary networks

Fabian Garmroudi[1,2,*], **Illia Serhiienko**[2], **Michael Parzer**[1], **Sanyukta Ghosh**[3], **Pawel Ziolkowski**[3], **Hieu Duy Nguyen**[4], **Cédric Bourgès**[2,5], **Yuya Hattori**[2], **Alexander Riss**[1], **Sebastian Steyrer**[1], **Gerda Rogl**[6], **Peter Rogl**[6], **Erhard Schafler**[7], **Naoyuki Kawamoto**[4], **Eckhard Müller**[3,8], **Ernst Bauer**[1], **Johannes de Boor**[3,9,*], **Takao Mori**[2,10,*]

[1] *Institute of Solid State Physics, TU Wien, 1040 Vienna, Austria*
[2] *International Center for Materials Nanoarchitectonics (WPI-MANA), National Institute for Materifals Science (NIMS), Tsukuba, Japan*
[3] *Institute of Materials Research, German Aeropspace Center (DLR), D-51147 Cologne, Germany*
[4] *Center for Basic Research on Materials (CBRM), NIMS, Tsukuba, Japan*
[5] *International Center for Young Scientists, National Institute for Materials Science (NIMS), Tsukuba, Japan*
[6] *Institute of Materials Chemistry, University of Vienna, 1090 Vienna, Austria*
[7] *Faculty of Physics, University of Vienna, 1090 Vienna, Austria*
[8] *Institute of Inorganic and Analytical Chemistry, Justus Liebig University Giessen, D-35392 Giessen, Germany*
[9] *University of Duisburg-Essen, Faculty of Engineering, Institute of Technology for Nanostructures (NST) and CENIDE, D-47057 Duisburg, Germany*
[10] *Graduate School of Pure and Applied Sciences, University of Tsukuba, Tsukuba, Japan*

Contents

1 Field-dependent Hall effect model **3**

2 Electronic band structure model and estimation of bipolar thermal conductivity **4**

2.1 Temperature-dependent electronic transport in the single-parabolic-band approximation 5
2.2 Multi-band and multi-carrier electronic transport 5
2.3 Modelling temperature-dependent transport in $Fe_2V_{0.95}Ta_{0.1}Al_{0.95}$ 6

*Correspondence to: f.garmroudi@gmx.at, Johannes.deBoor@dlr.de, mori.takao@nims.go.jp

3 Grain refinement via high-pressure torsion **7**
3.1 Attempts of reproducing high zT in $Fe_2V_{0.98}Ta_{0.1}Al_{0.92}$ 7
3.2 Investigation of HPT processing of $Fe_2V_{0.95}Ta_{0.05}Al_{0.9}Si_{0.1}$ and other optimized full-Heusler systems 7

4 Additional micro- and nanostructure analyses **8**
4.1 TEM investigation near the Heusler–Bi-Sb grain boundary 8
4.2 TEM investigation of a Heusler grain 9

5 Phonon band structures and densities of states **9**

6 Isotropy of thermal transport **10**

7 Thermal stability of composite samples **10**

8 Supplementary Figures **12**
8.1 HPT-deformed $Fe_2V_{0.98}Ta_{0.1}Al_{0.92}$: a comparison to literature 12
8.2 Effect of HPT on electronic transport in $Fe_2V_{0.95}Ta_{0.05}Al_{0.9}Si_{0.1}$ 13
8.3 Effect of HPT processing for different Heusler samples 14
8.4 Additional microstructure investigation of $Fe_2V_{0.95}Ta_{0.1}Al_{0.95}$ 15
8.5 Focused ion beam preparation of FVAB50 composite near a Heusler–$Bi_{1-x}Sb_x$ grain boundary 16
8.6 Additional TEM investigation of a FIB-fabricated sample near the Heusler–$Bi_{1-x}Sb_x$ grain boundary 17
8.7 Additional high-resolution TEM of Heusler grains in FVAB50 18
8.8 EDX spectroscopy of nanoscale precipitates 19
8.9 Additional EDX analysis of two-phase microstructures 20
8.10 Additional SEM analysis of FVABX composites 21
8.11 Quantitative analysis of Bi/Sb concentrations in FVABX composites 22
8.12 X-ray powder diffraction patterns 23
8.13 Comparison of the temperature-dependent Seebeck coefficient of polycrystalline $Bi_{0.9}Sb_{0.1}$ with literature 24
8.14 Constructing an effective band structure model for $Fe_2V_{0.95}Ta_{0.1}Al_{0.95}$ 25
8.15 Temperature-dependent Seebeck modelling of $Bi_{0.9}Sb_{0.1}$ and $Fe_2V_{0.95}Ta_{0.1}Al_{0.95}$ starting materials 26
8.16 Effective medium theory predictions of dimensionless figure of merit 27
8.17 Additional magneto-transport data of FVAB50 28
8.18 Anomalous Hall effect of non-magnetic FVAB50 compared to other magnetic materials 29
8.19 Consistency check of temperature-dependent electrical resistivity 30
8.20 Phonon dispersion and density of states mismatch 31
8.21 Comparison of thermal conductivity in different directions of the sample . . 32
8.22 Thermal stability of FVAB50 composite 33
8.23 Comparison of weighted mobilities with other material classes 34

1. Details of field-dependent Hall effect modelling

In a two-channel transport model for two types of charge carriers with charge $q_{1,2}$, carrier density $n_{1,2}$ and carrier mobility $\mu_{1,2}$, the field-dependent longitudinal resistivity $\rho_{xx}(B)$ and Hall resistivity $\rho_{xy}(B)$ can be expressed as[1]

$$\rho_{xx}(B) = \frac{q_1\, n_1\, \mu_1 + q_2\, n_2\, \mu_2 + (q_1\, n_1\, \mu_2 + q_2\, n_2\, \mu_1)\mu_1\, \mu_2\, B^2}{(q_1\, n_1\, \mu_1 + q_2\, n_2\, \mu_2)^2 + (q_1\, n_1 + q_2\, n_2)^2\, \mu_1^2\, \mu_2^2\, B^2} \tag{1}$$

and

$$\rho_{xy}(B) = \frac{q_1\, n_1\, \mu_1^2 + q_2\, n_2\, \mu_2^2 + (q_1\, n_1 + q_2\, n_2)^2\, \mu_1^2\, \mu_2^2\, B^2}{(q_1\, n_1\, \mu_1 + q_2\, n_2\, \mu_2)^2 + (q_1\, n_1 + q_2\, n_2)^2\, \mu_1^2\, \mu_2^2\, B^2}\, B\,. \tag{2}$$

From the above equations, it becomes obvious that non-linearities can occur in the field-dependent Hall resistivity. While previous models have employed four different fit parameters, i. e. the respective carrier densities and mobilities to model non-linear field dependencies, Eguchi and Paschen previously suggested a novel, more robust scheme for analyzing field-dependent magneto-transport properties[2]. This can be achieved by defining two new variables

$$N \equiv \frac{n_1 - n_2}{n_1 + n_2} \tag{3}$$

and

$$M \equiv \frac{\mu_1 - \mu_2}{\mu_1 + \mu_2}\,. \tag{4}$$

This allows rewriting Equation 1 and Equation 2 to the following new expressions, which depend on two, instead of four parameters

$$\begin{aligned}\rho_{xx}(B) = {}& \frac{2}{q_1\, n_+\, \mu_+} \\ & \times \frac{(1+N)(1+M) + \delta q(1-N)(1-M)}{[(1+N)(1+M) + \delta q(1-N)(1-M)]^2 + [(1+N) + \delta q(1-N)]^2(1-M^2)^2(\mu_+\, B)^2} \\ & + \frac{2}{q_1\, n_+\, \mu_+} \\ & \times \frac{[(1+N)(1-M) + \delta q(1-N)(1+M)](1-M)^2(\mu_+\, B)^2}{[(1+N)(1+M) + \delta q(1-N)(1-M)]^2 + [(1+N) + \delta q(1-N)]^2(1-M^2)^2(\mu_+\, B)^2}\end{aligned} \tag{5}$$

and

$$\begin{aligned}\rho_{xy}(B) = {}& \frac{2}{q_1\, n_+} \\ & \times \frac{(1+N)(1+M)^2 + \delta q(1-N)(1-M)^2 + [(1+N) + \delta q(1-N)](1-M)^2(\mu_+\, B)^2}{[(1+N)(1+M) + \delta q(1-N)(1-M)]^2 + [(1+N) + \delta q(1-N)]^2(1-M^2)^2(\mu_+\, B)^2}\, B\,,\end{aligned} \tag{6}$$

with the auxiliary variables $\delta q \equiv q_2/q_1$ and n_+ and μ_+ defined as

$$n_+ \equiv \frac{2}{q_1\,R_\mathrm{H}}\left[\frac{(1+N)(1+M)^2+\delta q(1-N)(1-M)^2}{[(1+N)(1+M)+\delta q(1-N)(1-M)]^2}\right] \tag{7}$$

and

$$\mu_+ \equiv \mu_\mathrm{H}\frac{(1+N)(1+M)+\delta q(1-N)(1-M)}{(1+N)(1+M)^2+\delta q(1-N)(1-M)^2}\,. \tag{8}$$

Here, R_H and μ_H are the Hall coefficient and Hall mobility in the linear response regime, i.e. $R_\mathrm{H} = \lim_{B\to 0}\rho_{xy}/B$ and $\mu_\mathrm{H} = \lim_{B\to 0}\rho_{xy}/(\rho_{xx}\,B)$, which can be obtained directly by performing a linear fit of the low-field experimental data.

Equation 6 was used to fit the experimental field-dependent ρ_{xy} data of the FVAB50 composite (Fig. 5b in the main manuscript) and to thereby extrac the single-channel Hall mobilities $\mu_{1,2}$ and carrier concentrations $n_{1,2}$. As an additional consistency check and to confirm whether the fitted parameters are based on solid grounds, we compared the zero-field electrical resistivity $\rho_{xx}(T)$ obtained from temperature-dependent measurements with $\rho_{xx}(T)$ obtained when recalculating the resistivity from the obtained $\mu_{1,2}$ and $n_{1,2}$ fit parameters $\rho_{xx} = (e\,n_1\,\mu_1 + e\,n_2\,\mu_2)^{-1}$. Fig. S19 shows this "consistency check" and reveals that the recalculated zero-field resistivity is in very good agreement with the temperature-dependent measurements, which further strenghtens the point that charge transport takes place via parallel conduction channels: (i) along the bulk $Fe_2V_{0.95}Ta_{0.1}Al_{0.95}$ matrix and (ii) along the $Bi_{1-x}Sb_x$ secondary phase at the grain boundaries.

2. Modelling temperature-dependent transport and estimating bipolar thermal conductivity

The temperature-dependent transport properties of the single-phase starting materials (putting aside the nanoscale Ta-rich precipitates in $Fe_2V_{0.95}Ta_{0.1}Al_{0.95}$) were modelled using the *SeeBand* code, which is a recently developed powerful fitting algorithm based on the Boltzmann transport theory and the parabolic band approximation [3]. In semiclassical Boltzmann theory and in the isotropical case of spherical Fermi surfaces, the generalized transport coefficients $L_\alpha(\mu, T)$ can be written as

$$L_\alpha(\mu,T) = e^2\int_{-\infty}^{\infty} D(E)v^2(E)\tau(E)(E-\mu)^\alpha\left(-\frac{\partial f}{\partial E}\right)dE\,. \tag{9}$$

with e being the elementary charge and $D(E)$, $v(E)$ and $\tau(E)$ being the electronic density of states, group velocity and relaxation time, respectively. The electrical conductivity, the Seebeck coefficient and the electronic part of the thermal conductivity are, for instance, directly determined by the transport coefficients in Equation 9 via the following expressions

$$\sigma = L_0(\mu,T)\,, \tag{10}$$

$$S = \frac{L_1(\mu, T)}{T\, L_0(\mu, T)}\,, \tag{11}$$

and

$$\kappa_{\mathrm{e}} = \frac{L_0(\mu, T)L_2(\mu, T) - L_1(\mu, T)^2}{T\, L_0(\mu, T)}\,. \tag{12}$$

2.1. Temperature-dependent electronic transport in the single-parabolic-band approximation

For parabolic bands it follows that $D(E) \propto E^{1/2}$, $v^2(E) \propto E$ and $\tau(E) \propto E^{-1/2}$ (when acoustic phonon and alloy disorder scattering are the dominant scattering processes) can be expressed as mere power-law expressions of the absolute energy. This allows one to define the temperature-dependent transport properties in terms of so-called Fermi integrals

$$F_j(\eta(T)) = \int_0^\infty \frac{\xi^j}{e^{\xi-\eta}+1} d\xi\,. \tag{13}$$

Here, $\xi = E/(k_{\mathrm{B}}T)$ and $\eta = \mu/(k_{\mathrm{B}}T)$ represent the reduced energy and chemical potential, respectively. The temperature-dependent electrical conductivity, Seebeck coefficient and Hall coefficient, which can be simultaneously fitted in the *SeeBand* algorithm, can be most generally written for a single parabolic band as

$$S(T) = \frac{k_{\mathrm{B}}}{e}\left[\eta - \frac{2F_1(\eta)}{F_0(\eta)}\right]\,, \tag{14}$$

$$\rho(T) = \left[\frac{2\sqrt{2}e^2\, k_{\mathrm{B}}T}{\pi^2\hbar^3}\left(\frac{\tilde{\tau}}{m}\right)T^{\gamma}F_0\left(\eta\right)\right]^{-1}\,, \tag{15}$$

$$R_{\mathrm{H}}(T) = \frac{\pi^2\hbar^3}{2e\left(2\, m\, k_{\mathrm{B}}T\right)^{3/2}}\frac{F_{-1/2}\left(\eta\right)}{F_0^2\left(\eta\right)}\,. \tag{16}$$

Here, $\tilde{\tau}$ represents a reduced electron relaxation time, which depends for instance on the acoustic deformation or alloy disorder scattering potentials, m is the band effective mass and γ is a scattering-specific parameter which is -1 and 0 for acoustic phonon and alloy disorder scattering, respectively.

2.2. Multi-band and multi-carrier electronic transport

In the case that two or more parabolic bands end up contributing to the transport properties, the single-band contributions have to be weighted and summed up appropriately[4,5] by the following relations

$$\sigma = \sum_i \sigma_i\,, \tag{17}$$

$$S = \frac{\sum_i S_i\sigma_i}{\sum_i \sigma_i}\,, \tag{18}$$

$$R_{\mathrm{H}} = \frac{\sum_i R_{\mathrm{H},i}\sigma_i^2}{(\sum_i \sigma_i)^2}\,, \tag{19}$$

and

$$\kappa_{\mathrm{e}} = T\sum_i L_i\sigma_i + T\left(\sum_i S_i^2\sigma_i - \frac{(\sum_i S_i\sigma_i)^2}{\sum_i \sigma_i}\right)\,. \tag{20}$$

In the above described theoretical framework, this leaves three independent fit parameters for the Seebeck coefficient in a two-band model, namely, the position of the Fermi energy with respect to the valence band edge E_{F}, the band gap or band overlap, i.e. the position of the conduction band bottom with respect to the valence band edge E_{g} as well as a conductivity weighting parameter $\epsilon_m = (m_1/m_2)^{-1}(\tau_1/\tau_2)(N_1/N_2)$, which includes the effective masses m_i, scattering times τ_i and degeneracies N_i of the two bands.

For the resistivity/conductivity, one additional fit parameter emerges, namely, the absolute scattering time of the valence band; the absolute scattering time of the conduction band can be calculated via the weighting parameter obtained during the Seebeck fit. Note that when two scattering processes, such as acoustic phonon and alloy disorder scattering are considered simultaneously, two additional fit parameters (the alloy disorder scattering potentials of the two respective bands) have to be introduced.

The Hall coefficient, allows for obtaining the absolute value of the effective masses in a final single-parameter fit. As described in Ref. [3], the ***SeeBand*** algorithm concurrently handles temperature-dependent data of the electrical resistivity, the Seebeck coefficient and the Hall coefficient for highly efficient and robust analyses of temperature-dependent transport and in order to deduce microscopic parameters regarding the electronic band structure and charge carrier scattering processes.

2.3. Modelling temperature-dependent transport in $Fe_2V_{0.95}Ta_{0.1}Al_{0.95}$

In Fig. S14, we show results of temperature-dependent analyses of all relevant transport properties for the Heusler material $Fe_2V_{0.95}Ta_{0.1}Al_{0.95}$, which has been used as a starting material for the high-performance composites. It can be seen that the Seebeck coefficient, the resistivity and the Hall coefficient can be simultaneously very well described using the very same band structure parameters, which hints at highly robust and reliable fitting results. These analyses reveal a narrow band gap of about 90 meV and rather heavy effective band masses for the conduction and valence band ($m_e = 8.5\,m_0$ and $m_h = 9.7\,m_0$). The latter most likely can be explained by the fact that the band structure of Fe_2VAl is highly complex when E_{F} is shifted from within the pseudogap – where mostly two bands (one at Γ and one at X) dominate the transport – into either the conduction or the valence band, where highly degenerate and non-parabolic bands are present. This is reflected also in a steep slope of the density of states at both the conduction and valence band side. Since the present sample is heavily doped ($n_{\mathrm{H}} = 1.8 \times 10^{21}\,\mathrm{cm}^{-3}$) with E_{F} shifted deep into the conduction band states, we expect these non-parabolic bands to contribute to the transport as well.

Nonetheless, the fact that all transport properties can be simultaneously well described by this *effective* band structure model, allows us to visualize the individual contributions of the

hole- and electron-like carriers to the total Seebeck coefficient (see Fig. S14d) and estimate the bipolar contribution to the electronic thermal conductivity (see Fig. S14e), the latter of which is crucial for determining the real value of the lattice thermal conductivity. Indeed, it can be seen in Fig. S14e, that the Wiedemann-Franz law insufficiently and inadequately captures the electronic contribution to the thermal conductivity at high temperatures. For instance, at $\approx 500\,\mathrm{K}$, the bipolar term is already twice as large as compared to the Wiedemann-Franz term. However, Fig. S14f, where the theoretical upper limit of the dimensionless figure of merit for $\kappa_\mathrm{L} \to 0$ is plotted and compared to experimental findings, clearly demonstrates that still lattice-dominated heat transport severely hampers the overall performance of these systems and there is significant room for improvement in terms of reducing κ_L, especially when a high weighted mobility can be retained.

3. Grain refinement and severe plastic deformation via high-pressure torsion

3.1. Attempts of reproducing high zT in $Fe_2V_{0.98}Ta_{0.1}Al_{0.92}$

In 2018, Masuda et al. reported on the effect of high-pressure torsion (HPT) on the microstructure and thermoelectric properties of Fe_2VAl-based full-Heusler compounds, achieving enhancements of the dimensionless figure of merit up to $zT = 0.3$ at around $500\,\mathrm{K}$[6]. These enhancements were attributed to a reduction of the grain size during severe plastic deformation via HPT. Processing of thermoelectric materials via HPT has also been very effectively employed for skutterudites previously. For instance, Rogl et al. achieved an outstanding thermoelectric performance up to $zT > 2$ in multi-filled skutterudites[7–9]. On the other hand, the authors found much less impressive enhancements of zT for half-Heusler compounds, when HPT has been employed to refine the grain size and introduce various defects such as dislocations[10,11]. This can be ascribed to a strong tradeoff in carrier mobility and lattice thermal conductivity for the latter. In 2022, Fukuta and the same co-authors of Ref. [6], reported further enhancements of zT up to $zT = 0.37$ at around $400\,\mathrm{K}$ in $Fe_2V_{0.98}Ta_{0.1}Al_{0.92}$. These promising results, motivated us to (i) reproduce them and (ii) investigate other samples with different compositions to potentially realize even high thermoelectric performance. These experimental endeadvours are summarized in this section.

Fig. S1 shows a comparison of experimental data by Fukuta et al. and data obtained in this work by following exactly the same synthesis and HPT processing conditions mentioned in Ref. [12]. It can be seen that while the temperature-dependent Seebeck coefficient and thermal conductivity could be more or less well reproduced, the electrical resistivity found in the present study, is more than a factor of two larger than that reported in Ref. [12]. Consequently, we were not able to realize an enhancement of zT in the present case. In fact, zT was even smaller than for the sample which was not subjected to HPT processing. This highlights, that exact setup-specific processing conditions during HPT are likely very important to realize the desired microstructure and properties.

3.2. Investigation of HPT processing of $Fe_2V_{0.95}Ta_{0.05}Al_{0.9}Si_{0.1}$ and other optimized full-Heusler systems

In order to determine whether enhancements of zT might be realizable for different Fe_2VAl-based Heusler compounds, we investigated a variety of samples with promising electronic properties (high power factor and weighted mobiltiy) before and after HPT processing. Fig. S2 summarizes the evolution of the electronic transport properties ($S(T)$, $\rho(T)$ and $PF(T)$) of the recently discovered ultrahigh-PF compound $Fe_2V_{0.95}Ta_{0.05}Al_{0.9}Si_{0.1}$ [13]. Again, similar to the afore-mentioned case of $Fe_2V_{0.98}Ta_{0.1}Al_{0.92}$, we observe a significant (factor 4) increase in the electrical resistivity, which persists even after several measurement-induced annealing cycles (see Fig. S2a). Simultaneously, the Seebeck coefficient decreases as a result of mechanically induced antisite disorder, which was confirmed by emergent magnetic behavior of the samples (not shown here). Fig. S2c and Fig. S2d show that the electrical resistivity progressively increases and the power factor progressively decreases as the number of revolutions during HPT increases. Thus, HPT turns out to be highly detrimental to the electronic transport properties, hindering improvements in the figure of merit. Virtually the same trend has been observed for all samples investigated and no enhancement of zT was realized even when the number of revolutions or the processing temperature during HPT were varied. Fig. S3 highlights that zT decreases after HPT as compared to the non-processed reference samples, pointing at a detrimental tradeoff between carrier weighted mobility (electronic scattering) and lattice thermal conductivity (phonon scattering). Going into the future, it will be essential to indentify how to overcome this tradeoff and whether other groups are able to reproduce initial enhancements observed in Refs. [6,12].

4. Additional micro- and nanostructure analyses of FVABX composites

4.1. TEM investigation near the Heusler–Bi-Sb grain boundary

Figure S6 illustrates TEM images acquired near the grain boundary (GB) of a FIB-fabricated sample. Panel (a) displays a bright-field image where the Fe_2VAl grain and the Bi-Sb grain are separated by the GB. Panel (b) shows a lattice image near the GB, while panel (c) presents the FFT of the area limited by the yellow square. Panel (d) depicts a simulation of Selected Area Diffraction (SAD) result adopting the ReciPro software. Panels (e) and (f) demonstrate an inverse FFT of panel (c), choosing spots 022 and $\overline{022}$ for panel (e) and spots $\overline{1}\overline{1}\overline{1}$ and $1\overline{1}1\overline{1}$ for panel (f), respectively. Interplanar distances are identified by measuring distances between successive fringes; distances between 10 successive fringes are measured and displayed in panels (e) and (f). Panel (g) provides a low-magnification TEM image of an area including Fe_2VAl and Bi-Sb grains, along with a Ta-rich precipitate. Panel (h) shown a lattice image of the Ta-rich precipitate acquired in the area marked by the yellow square in panel (g), showing an interplanar distance of approximately 6.4 Å, which is a twofold difference compared to that of the Fe_2VAl phase. Panels (i) and (k) are SAD patterns acquired at the Ta-rich precipitate and Fe_2VAl grain, respectively, along the [211] zone-axis. These figures indicate that the Ta-rich precipitate has a superstructure of the Heusler phase with a nearly similar crystal orientation, suggesting a relationship in the formation of the

Fe_2VAl phase and the Ta-rich precipitate. A light ring, indicated by a yellow arrow in panel (i), is attributed to an amorphous phase, likely covering the Ta-rich precipitate. Panel (l) shows a lattice image of one of the nanograins, whose phases are unidentified, observed inside the $Bi_{1-x}Sb_x$ grain and near the Ta-rich precipitate, marked by the blue dashed ellipse in panel (h).

4.2. TEM investigation of a Heusler grain

Figure S7 presents Transmission Electron Microscopy (TEM) images of a powder sample, which was obtained by crushing a sintered sample. Panel (a) shows a lattice image of one of the grains, highlighting its atomic arrangement. A more detailed view is provided in panel (b), which displays a high-magnification image of the region outlined by the yellow square in panel (a). Here, interplanar distances of approximately 2.0 Å, characteristic of the Fe_2VAl phase, are observed. The corresponding fast Fourier transform (FFT) of this region is shown in panel (c), providing insight into the crystallographic order of the grain. Panel (d) presents a simulation of the selected area diffraction (SAD) pattern along the [100] zone-axis, offering further confirmation of the crystal structure. In panel (e), another grain is displayed with its atomic lattice clearly visible. A high-magnification image of the yellow-square region from panel (e) is depicted in panel (f), showing interplanar distances of approximately 3.3 Å for the Fe_2VAl phase. The FFT of this region is provided in panel (g), while panel (h) reveals an inverse FFT of panel (g). The green circles in panel (g) mark specific spots in the FFT, indicating several dislocation defects, which are made more evident in the inverse FFT image in panel (h). These defects are of particular interest for understanding the local structural characteristics of the Fe_2VAl phase.

5. Acoustic mismatch in the phonon band structures and densities of states of elemental Bi and Heusler-type Fe_2VAl

Fig. S20 compares phonon band structures and densities of states of Heusler-type Fe_2VAl and elemental Bi. The strong mismatch in the acoustic properties is reflected in a significantly flatted phonon dispersion for Bi when compared to Fe_2VAl. This can be mainly attributed to the much higher atomic mass of Bi compared to Fe, V and Al. Indeed, as follows already in a simple model for a one-dimensional monoatomic chain, the dispersion relation $\omega(k)$ can be expressed as

$$\omega(k) = 2\sqrt{\frac{K}{m}} \sin\left(\frac{1}{2}ka\right) . \tag{21}$$

Here, K represents a spring force constant acting on the atoms, m the atomic mass and a the interatomic spacing. While the acoustic branches of Fe_2VAl reach up to about 9 THz and the highest lying optical branches reach a bit more than 12 THz, the phonon spectrum of elemental Bi is capped at frequencies less than 4 THz. This leads to an ≈ 9 THz mismatch in the phonon densities of states of the two respective materials. Although substitution with Ta in $Fe_2V_{1-x}Ta_xAl$ or Sb in $Bi_{1-x}Sb_x$ could slightly change the phonon spectra (W substitution

in $Fe_2V_{1-x}W_xAl$ has been reported to lead to a 6 and 10 % reduction of the sound velocities for $x = 0.125$ and $x = 0.25$, respectively[14]), the qualitatively large difference between the two chemically distinct materials is expected to be the same. This phonon mismatch is also reflected in substantially different Debye temperatures and sound velocities (see also Fig. S20c): $\Theta_D \approx 120\,K\,/\,v_l \approx 2240\,m\,s^{-1}$ for elemental Bi and even lower $\Theta_D \approx 90\,K\,/\,v_l \approx 1990\,m\,s^{-1}$ for $Bi_{0.9}Sb_{0.1}$[15] but much higher $\Theta_D \approx 540\,K\,/\,v_l \approx 6380\,m\,s^{-1}$ for Fe_2VAl[16]. Such large mismatching phonon dispersions can be very effective in reducing lattice thermal conductivity in composite materials. The idea is that when a phonon crosses the heterogeneous interface between the two distinct materials, only phonons with matching densities of states are allowed to pass through, effectively filtering phonons in the mismatching energy spectrum. This leads to an extremely large thermal grain boundary resistance. The prospect of engineering such mismatching phonon modes in composite materials has been reported previously, e.g., in Cu_2Se–graphene[17], Bi_2Te_3–PEDOT:PSS[18] and other composite systems[19–21].

6. Isotropy of thermal transport confirmed by measurements in different directions of the sample

Commonly, in thermoelectric materials prepared via sintering, the thermal conductivity is measured parallel to the compaction direction, whereas the electronic transport properties (resistivity and Seebeck coefficient) are usually measured only perpendicular to the direction of compaction. This can lead to erroneous estimates of the dimensionless figure of merit when the material displays anisotropy in its microstructure or crystal structure, and thus, in its transport properties. For instance, Kim et al. reported in 2015 extraordinarily high values of the dimensionless figure of merit up to $zT = 1.86$ at 320 K in $(Bi, Sb)_2Te_3$ alloys prepared via liquid-phase sintering[22]. However, it was shown a couple of years later by Rigui et al. that these high zT values probably result erroneously from a favorable but incorrect combination of thermal conductivity measured along the compaction direction of a sintered sample with an anisotropic microstructure, while charge transport is measured in the direction perpendicular to the compaction direction[23].

To rule out any anisotropy of thermoelectric transport in our composite samples, we measured the thermal conductivity in different directions of the sample using different techniques (see Fig. S21). Excellent agreement between low-temperature measurements performed perpendicular to the pressing direction, using a steady-state method, and high-temperature measurements performed parallel to the pressing direction, using a laser flash diffusivity method, confirm that the microstructure of our samples does not display any sizeable anisotropy which might affect the thermal conductivity in a similar manner to that reported in Ref. [22].

7. Thermal stability of high-performance FVAB50 composite for several measurement cycles

To confirm that our approach yields thermodynamically stable composite samples, we measured the temperature-dependent thermoelectric properties of the sample with the highest

thermoelectric performance, discussed extensively within the main text, multiple times to ensure that there is no sizeable deviation in the properties between different thermal cycles. These results are plotted in Fig. S22. It can be seen that, at least up to approximately 500 K, there is no significant deviation and the measurement curves are consistent well within the uncertainty of the experimental setups, both in terms of absolute values and their temperature-dependent behavior. Thus, one may assume that the obtained two-phase microstructure is thermodynamically stable (or metastable with very large timescales of atomic diffusion) around room temperature, where zT peaks.

8. Supplementary Figures

8.1. HPT-deformed $Fe_2V_{0.98}Ta_{0.1}Al_{0.92}$: a comparison to literature

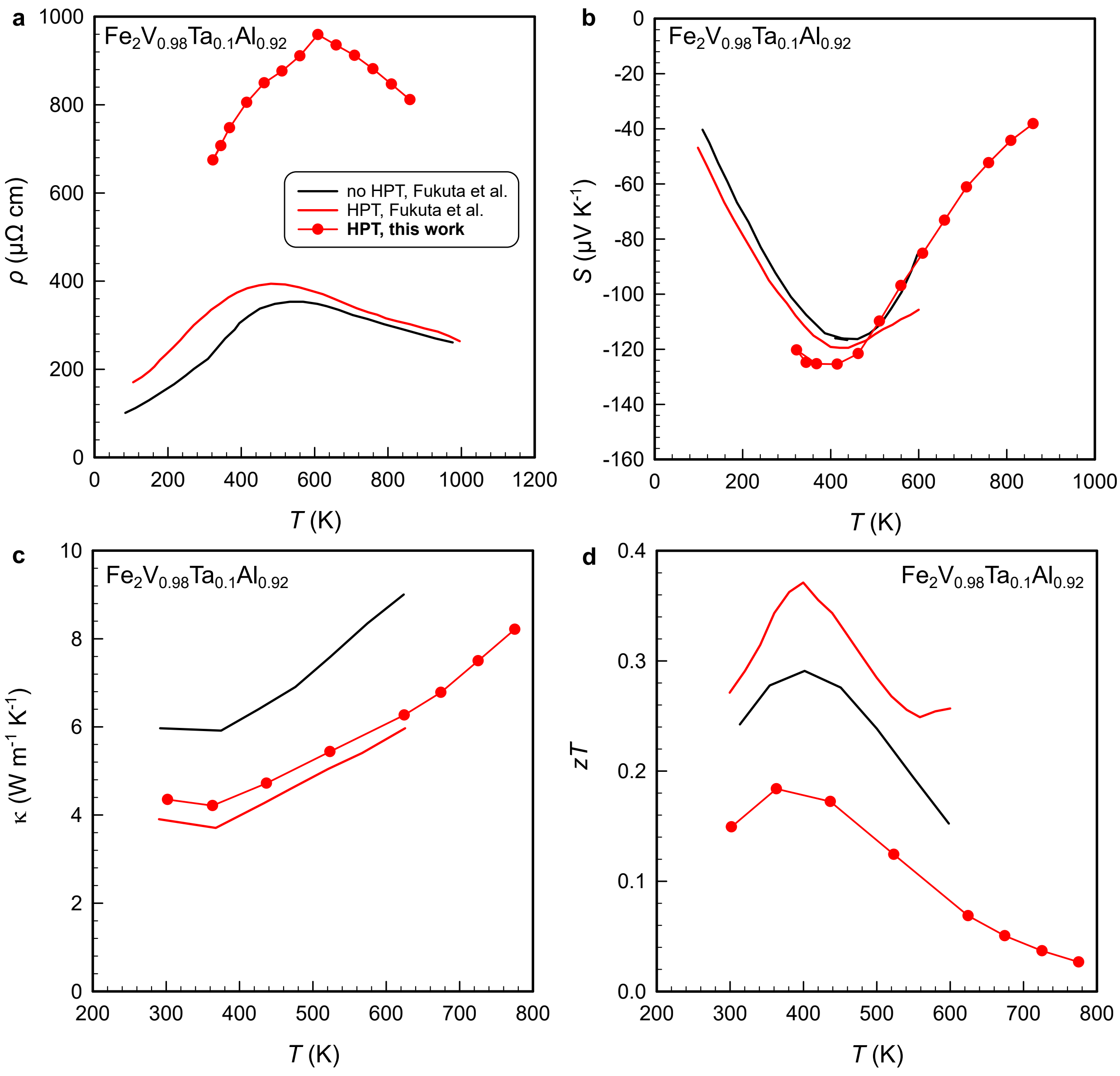

Fig. S1 | Thermoelectric properties of $Fe_2V_{0.98}Ta_{0.1}Al_{0.92}$ plastically deformed via high pressure torsion (HPT). **a**, Electrical resistivity, **b**, Seebeck coefficient, **c**, thermal conductivity and **d**, dimensionless figure of merit. Experimental data obtained within this study are compared to those by a recent study by Fukuta et al. [12]. While the Seebeck coefficient and thermal conductivity can be well reproduced, the electrical resistivity is significantly higher in this work, leading to an almost two times smaller zT.

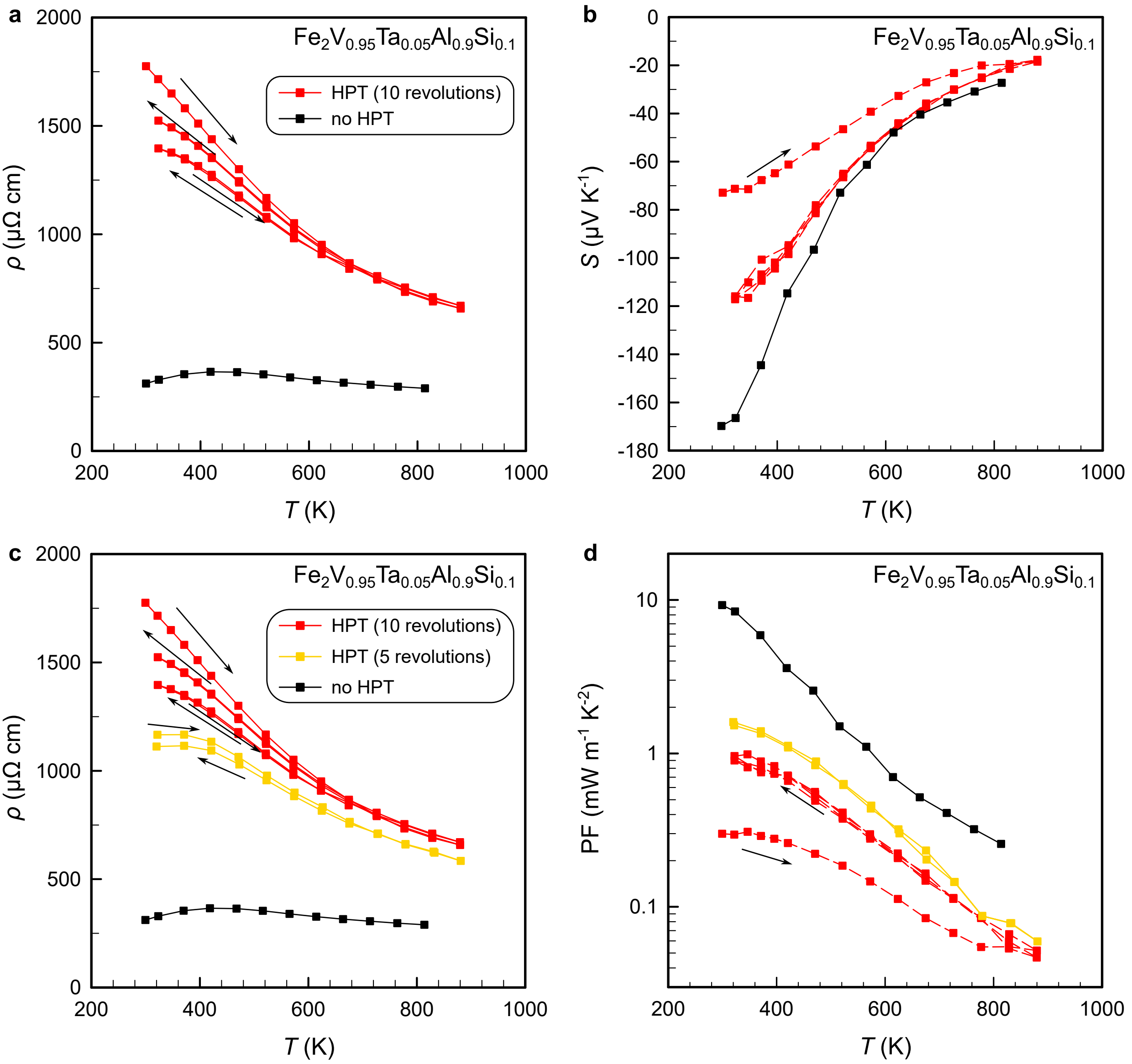

Fig. S2 | Effect of high-pressure torsion (HPT) on the thermoelectric properties of $Fe_2V_{0.95}Ta_{0.05}Al_{0.9}Si_{0.1}$. **a**, Temperature-dependent electrical resistivity and **b**, Seebeck coefficient of $Fe_2V_{0.95}Ta_{0.05}Al_{0.9}Si_{0.1}$ before and after HPT treatment with 10 revolutions. Several heating and cooling cycles are shown until the properties reach an equilibrium. **c**, Temperature-dependent electrical resistivity and **d**, power factor (PF $= S^2/\rho$) before and after HPT. The electrical resistivity increases substantially and PF decreases with the number of revolutions during HPT due to an increase in the defect density comprising atomic-scale antisite disorder, nano-scale discloations and grain size reduction.

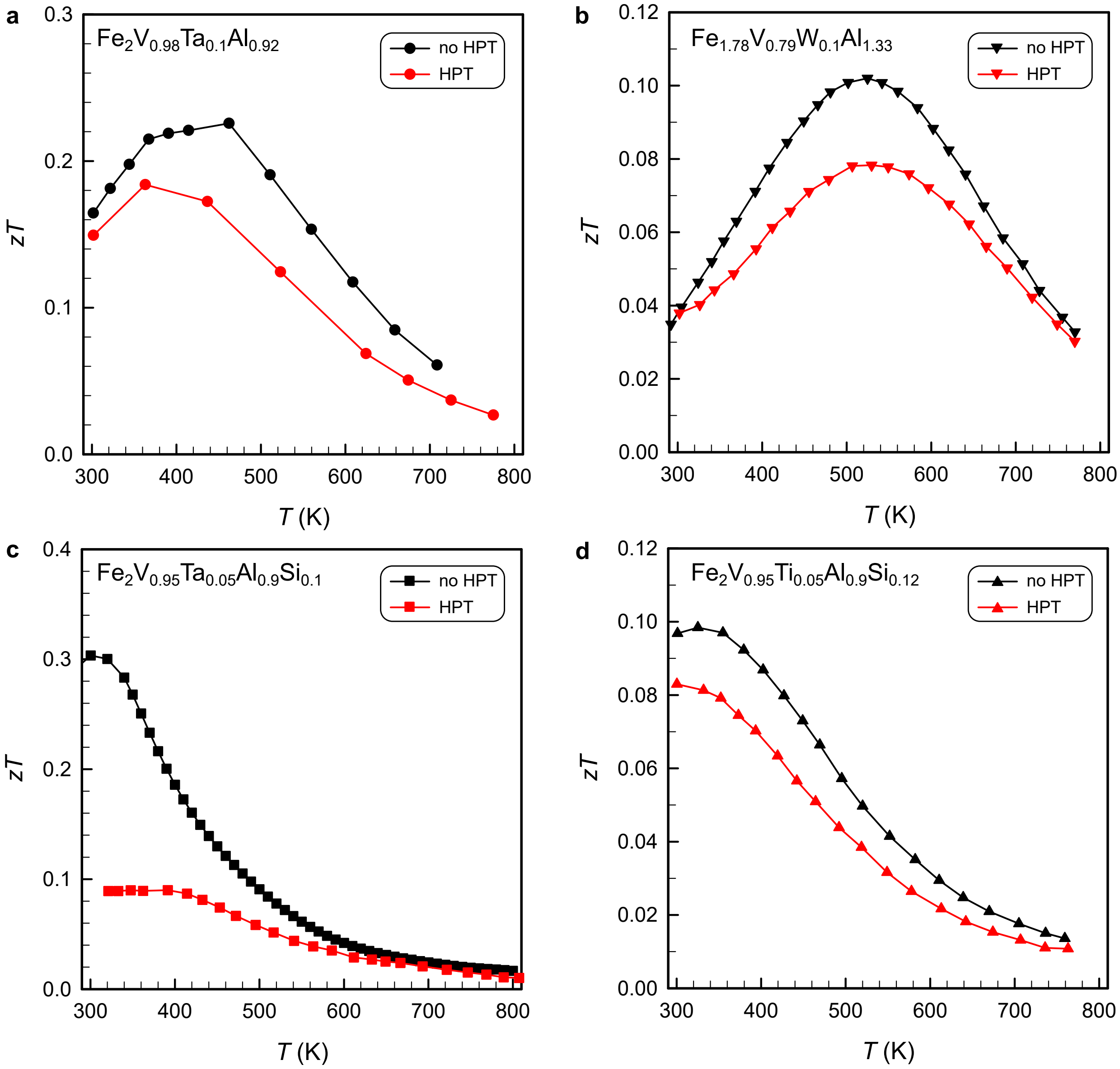

Fig. S3 | Comparison of dimensionless figure of merit of different Fe_2VAl-based full-Heusler compounds with optimized composition before and after HPT. Temperature-dependent figure of merit before and after HPT (10 revolutions) for **a**, off-stoichiometric and Ta-substituted $Fe_2V_{0.98}Ta_{0.1}Al_{0.92}$, **b**, off-stoichiometric and W-substituted $Fe_{1.78}V_{0.79}W_{0.1}Al_{1.33}$, **c**, Ta- and Si-co-substituted $Fe_2V_{0.95}Ta_{0.05}Al_{0.9}Si_{0.1}$ and **d**, Ti- and Si-co-substituted $Fe_2V_{0.95}Ti_{0.05}Al_{0.9}Si_{0.1}$.

8.4. Additional microstructure investigation of $Fe_2V_{0.95}Ta_{0.1}Al_{0.95}$

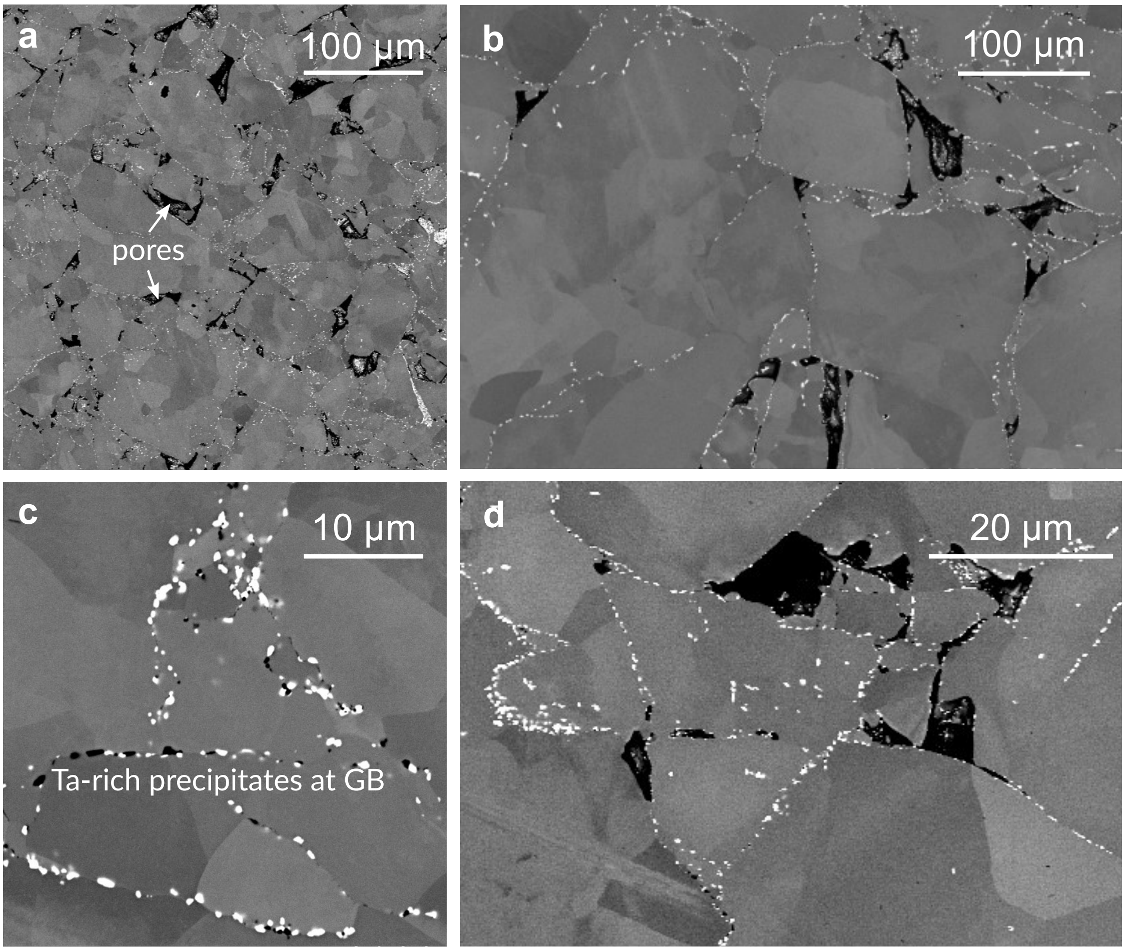

Fig. S4 | Microstructures of pristine $\mathbf{Fe_2V_{0.95}Ta_{0.1}Al_{0.95}}$ (FVAB0) probed by backscattered scanning electron microscopy. a,**b**, BS-SEM images at low magnification at different areas of the same sample reveal pores evenly distributed throughout the whole sample. **c**,**d**, High-magnification BS-SEM images at different areas of the same sample reveal small, Ta-rich precipitates which segregate at the grain boundaries, likely already during the melt-synthesis in the induction furnace and further upon annealing during the high-temperature sintering process.

8.5. Focused ion beam preparation of FVAB50 composite near a Heusler–$Bi_{1-x}Sb_x$ grain boundary

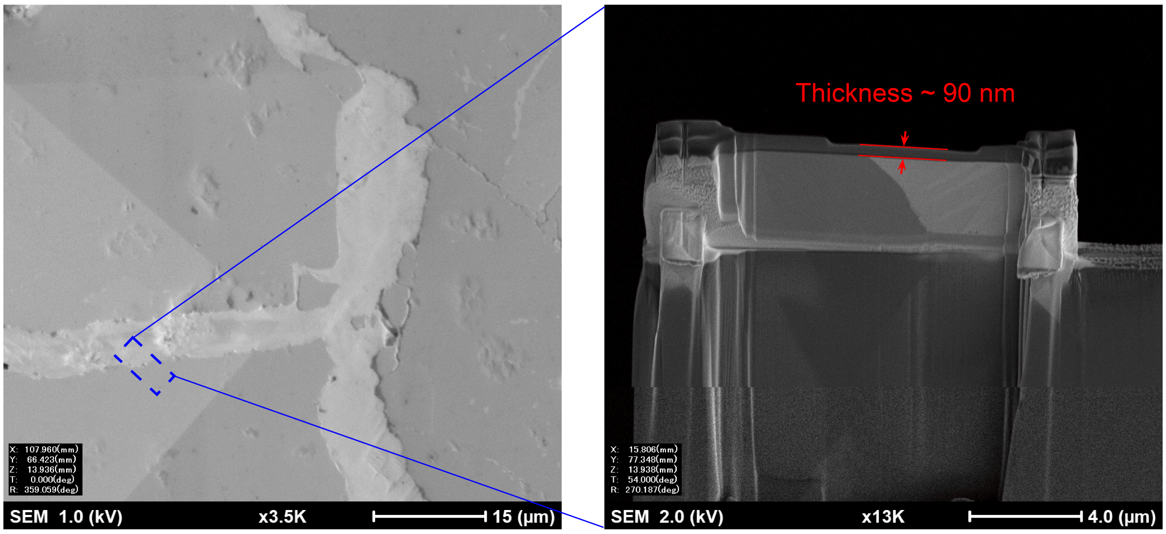

Fig. S5 | Focused ion beam perparation of a FVAB50 composite sample near the grain bounadry of the Heusler phase and $Bi_{1-x}Sb_x$.

8.6. *Additional TEM investigation of a FIB-fabricated sample near the Heusler–$Bi_{1-x}Sb_x$ grain boundary*

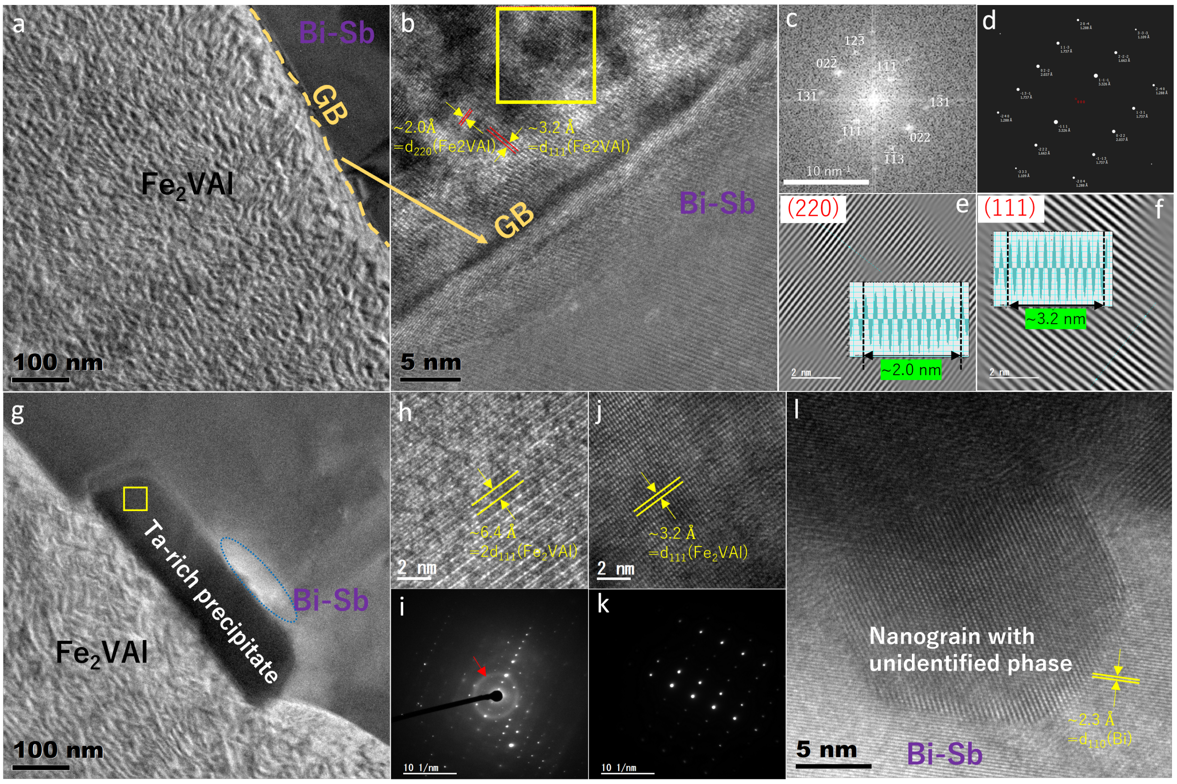

Fig. S6 | Transmission electron microscopy of an FIB-fabricated specimen near the grain boundary of the Heusler phase and $Bi_{1-x}Sb_x$. **a**, Bright-field image showing the Fe_2VAl grain and the Bi-Sb grain, separated by a grain boundary (GB). **b**, Lattice image taken near the GB. **c**, FFT of the region highlighted by the yellow square. **d**, Simulated Selected Area Diffraction (SAD) pattern using Recipro. **e** and **f**, Inverse FFT images derived from panel **c**, where panel **e** corresponds to the selection of spots 0–22 and 02–2, and panel **f** corresponds to spots –111 and 1–1–1. Interplanar distances are determined by measuring the spacing between successive fringes, with distances between 10 fringes shown in panels **e** and **f**. **g**, Low-magnification TEM image capturing Fe_2VAl and Bi-Sb grains, alongside a Ta-rich precipitate. **h**, Lattice image of the Ta-rich precipitate taken from the area marked by the yellow square in panel **g**, revealing an interplanar distance of approximately 6.4 Å, double that of the Fe_2VAl phase. **i** and **k**, SAD patterns obtained from the Ta-rich precipitate and Fe_2VAl grain, respectively, along the [211] zone-axis. These results indicate that the Ta-rich precipitate has a Heusler superstructure with nearly the same crystal orientation as the Fe_2VAl grain, suggesting a relationship in their formation. The light ring in panel **i**, marked by a red arrow, likely corresponds to an amorphous phase covering the Ta-rich precipitate. **l**, Lattice image of a nanograin (phase unidentified) within the Bi-Sb grain and near the Ta-rich precipitate, as indicated by the blue dashed ellipse in panel **g**.

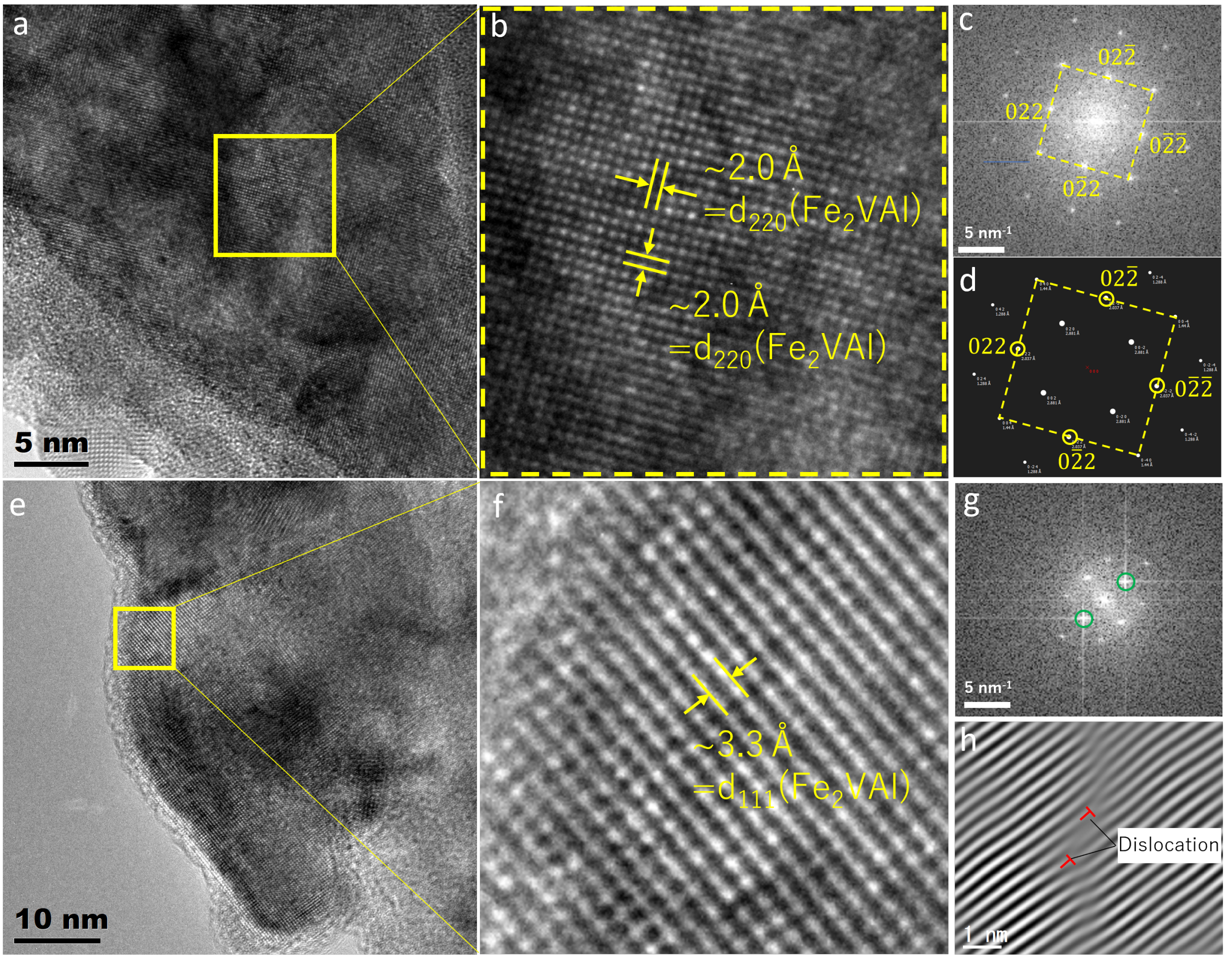

Fig. S7 | Transmission electron microscopy (TEM) of a grain of the FVAB50 composite conducted on powder of a crushed sintered sample. **a**, Lattice image of a grain of the Heusler phase. **b**, High-magnification image of the area delimited by a yellow square in **a**, revealing interplanar distances of approximately 2.0 Å of the Fe_2VAl phase. Panel **c** depicts the FFT of **b**. **d**, Simulation of the SAD pattern along the [100] zone-axis. **e**, Lattice image of another grain. **f**, High-magnification image of the area delimited by a yellow square in **e**, showing an interplanar distance of approximately 3.3 Å of the Fe_2VAl phase. Panel **g** shows the FFT of **f**. **h**, Inverse FFT of **g**, where spots marked by green circles in **g** indicate several dislocation defects.

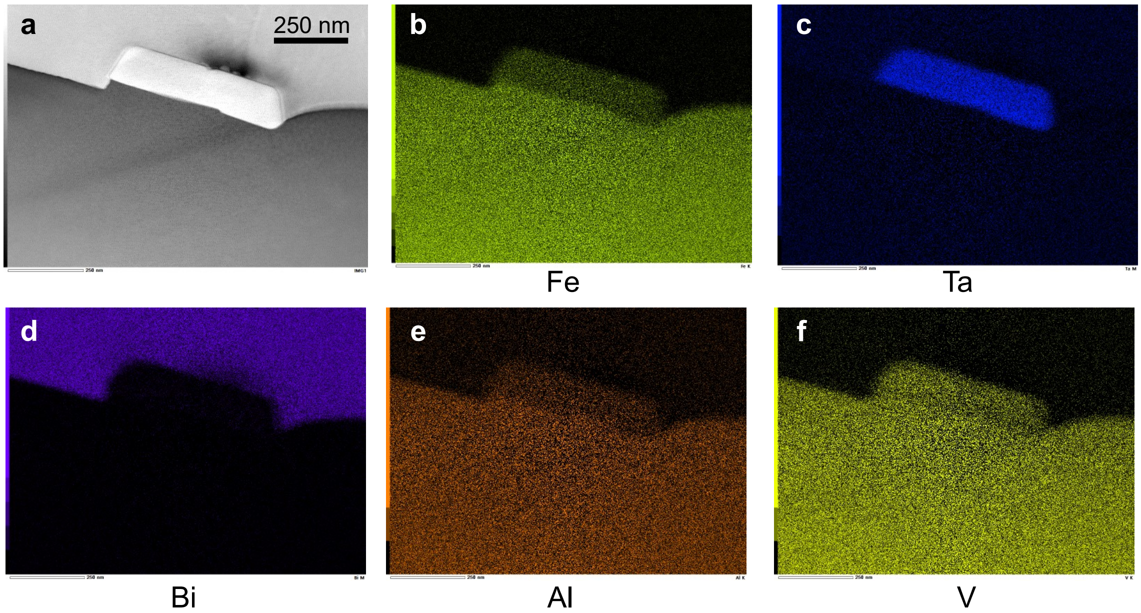

Fig. S8 | Energy-dispersive X-ray spectroscopy of a Ta-rich precipitate at the Heusler–$Bi_{1-x}Sb_x$ interface. **a**, STEM image **b-f**, EDX mapping of Fe, Ta, Bi, Al and V, respectively, reveals a precipitate which is rich in Ta but contains also all other elements of the Heusler phase, namely Fe, V and Al. The composition of the precipitate is obtained as 39.8 at.% Ta, 28.2 at.% Fe, 19.3 at.% V, 12.7 at.% Al.

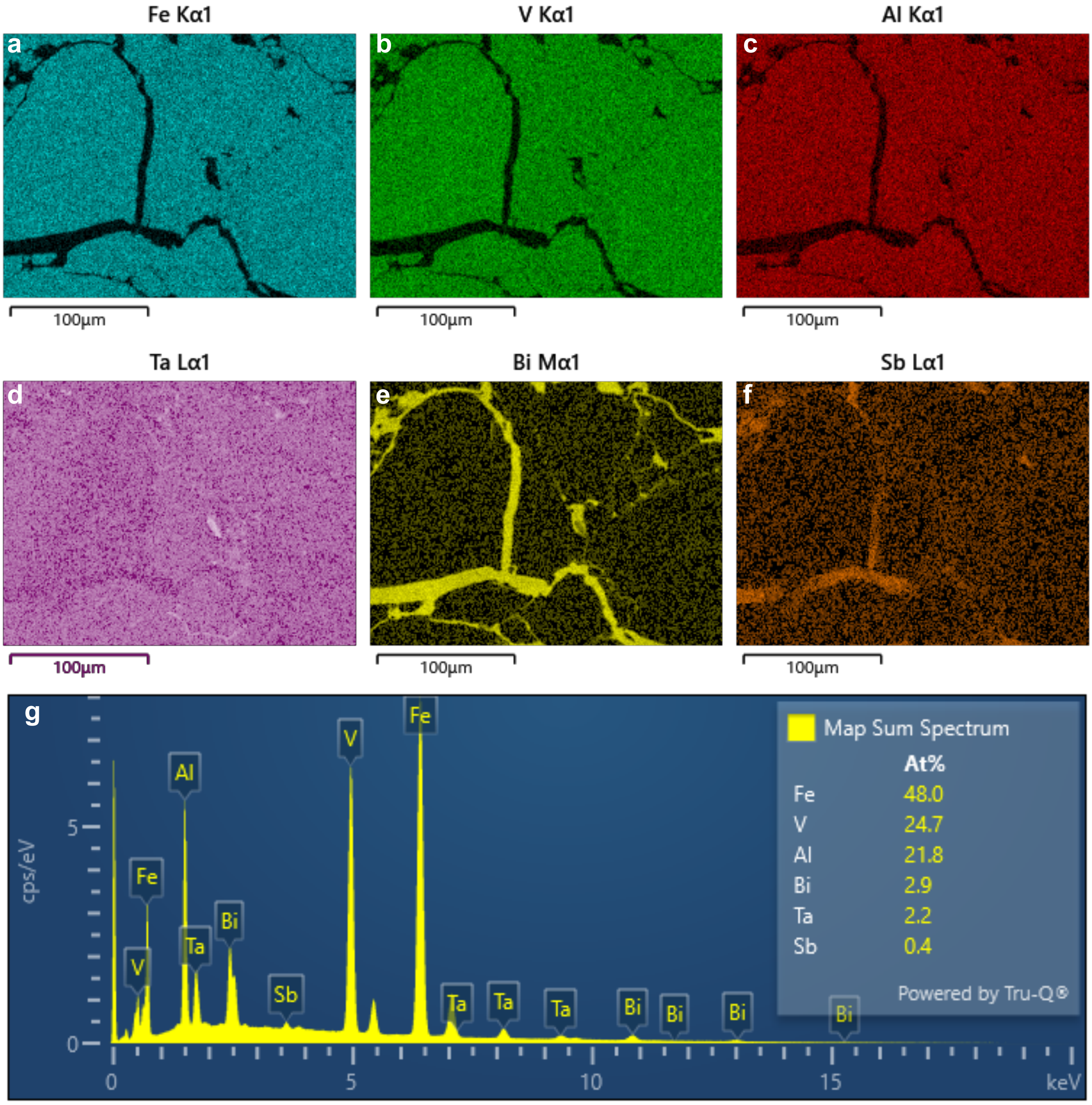

Fig. S9 | Additional energy-dispersive X-ray spectroscopy of FVABX composites. a-f Elemental mappings of Fe, V, Al, Ta, Bi and Sb, respectively. Variations in the Sb concentration within the $Bi_{1-x}Sb_x$ secondary phase are apparent and indicate that the composition of $Bi_{0.9}Sb_{0.1}$ changes during liquid-phase sintering and depends on the local surrounding in the sample. **g**, EDX spectrum with contributions of the different elements in atomic percent.

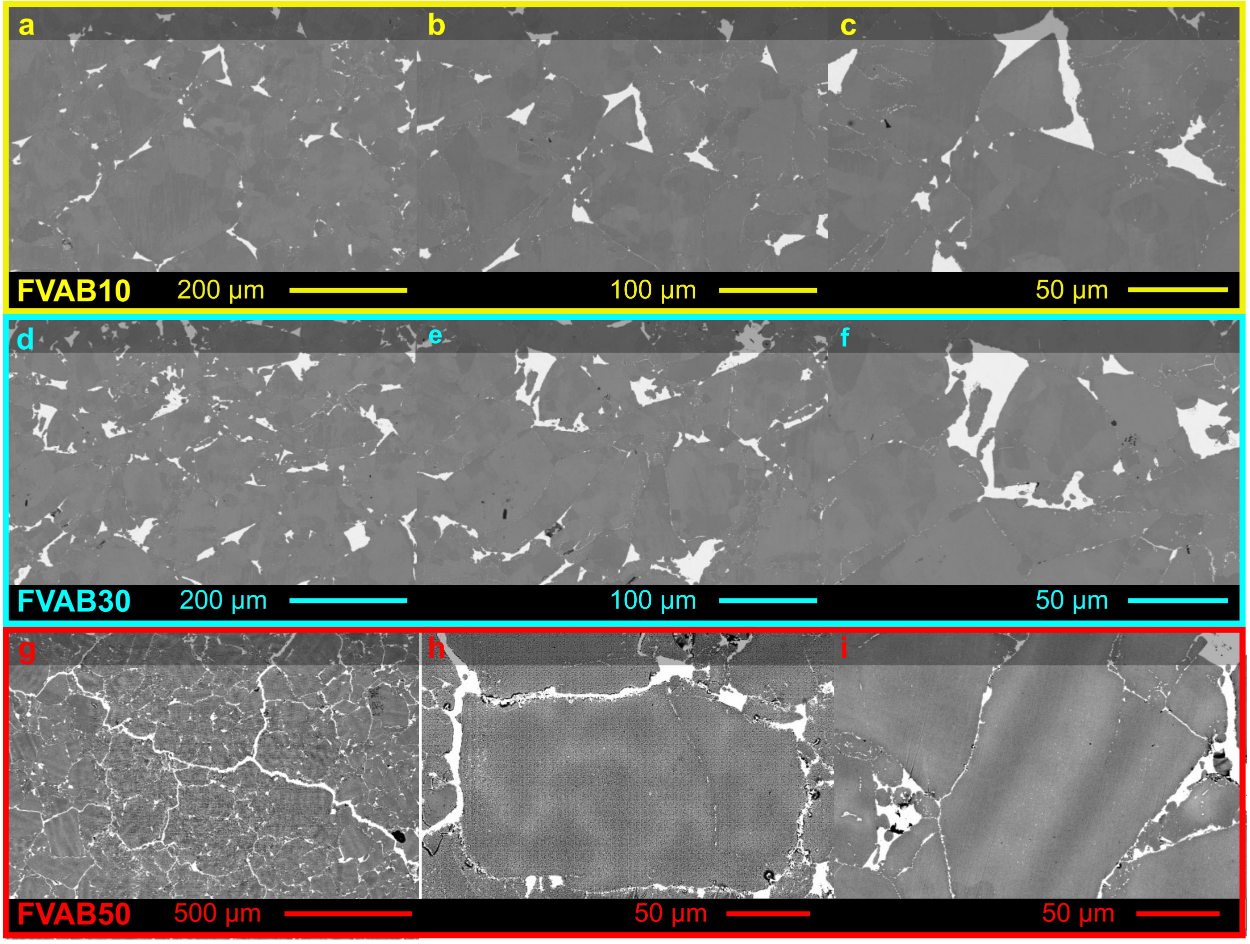

Fig. S10 | Additional backscattered electron micrographs of FVAB*X* composites. a-c, BSE-SEM images of FVAB10 (10 vol.% $Bi_{0.9}Sb_{0.1}$ added before sintering), **d-f**, FVAB30 (30 vol.% $Bi_{0.9}Sb_{0.1}$ added before sintering) and **g-i**, FVAB50 (50 vol.% $Bi_{0.9}Sb_{0.1}$ added before sintering), at different magnifications. It can be seen that, initially, only the triple junctions between the Heusler grains are filled with the secondary phase, whereas a coherent and connected network of $Bi_{1-x}Sb_x$ develops for the latter. Simultaneously, a patterned microscture, with brightness variations inside the Heusler grains corresponding to Ta-supersaturated regions, emerges.

8.11. Quantitative analysis of Bi/Sb concentrations in FVABX composites

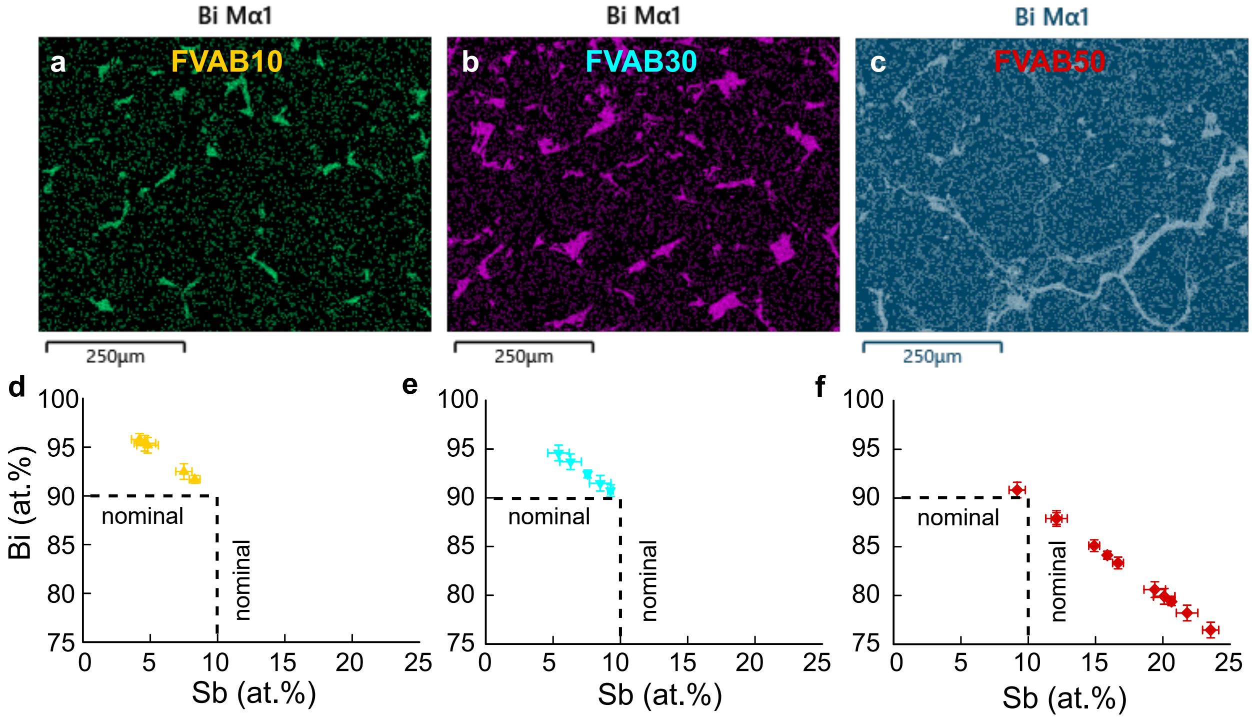

Fig. S11 | EDX mapping and composition analysis of the secondary phase. a-c, EDX mapping of Bi in FVAB10, FVAB30 and FVAB50 composites, respectively. **d-f** shows Bi and Sb concentrations obtained from EDX mapping and point analyses at various different spots in the sample. Interestingly, these results suggest that the Sb concentration increases when the amount of $Bi_{0.9}Sb_{0.1}$ powder added before sintering is increased. Moreover, the composition of the secondary phase fluctuates at the microscale throughout the sample (cf. also Fig. S9f).

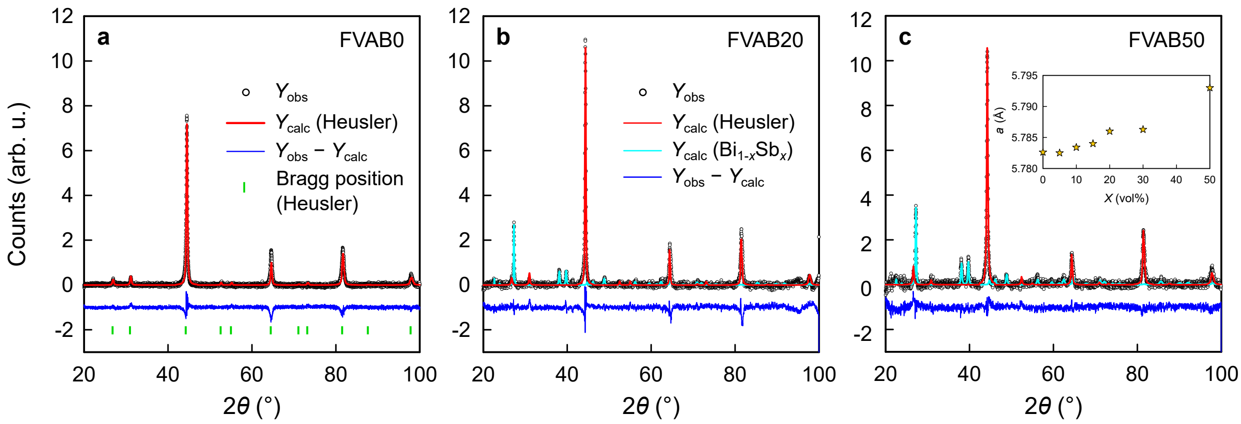

Fig. S12 | X-ray powder diffraction patterns of FVAB*X* composites. **a**, XRD pattern of the pure Heusler sample with the nominal composition $Fe_2V_{0.95}Ta_{0.1}Al_{0.95}$. Red solid line shows a Rietveld refinement, from which the lattice parameter was derived as $a = 0.578(4)$ nm. **b**, XRD pattern for the FVAB20 composite ($Fe_2V_{0.95}Ta_{0.1}Al_{0.95}$ + 20 vol.% $Bi_{0.9}Sb_{0.1}$ added before sintering). Red and cyan solid lines correspond to the Rietveld refinements of the Heusler and Bi-Sb phase, respectively. **c** XRD pattern and refinements for the high-performance FVAB50 composite sample, which was prepared via liquid phase sintering by adding 50 vol.% $Bi_{0.9}Sb_{0.1}$ powder before the compaction process. Inset shows the room-temperature lattice parameter of the Heusler phase as a function of the $Bi_{0.9}Sb_{0.1}$ concentration added before sintering.

8.13. Comparison of the temperature-dependent Seebeck coefficient of polycrystalline $Bi_{0.9}Sb_{0.1}$ with literature

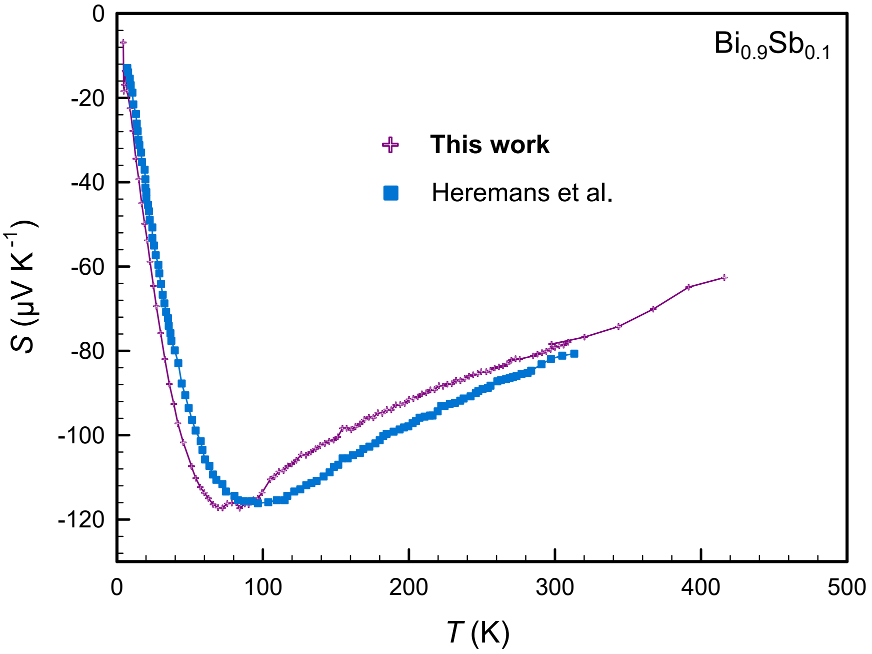

Fig. S13 | Consistency check with previous work. Comparison of the temperature-dependent Seebeck coefficient of polycrystalline $Bi_{0.9}Sb_{0.1}$ from this study and data obtained previously by Heremans et al. [24].

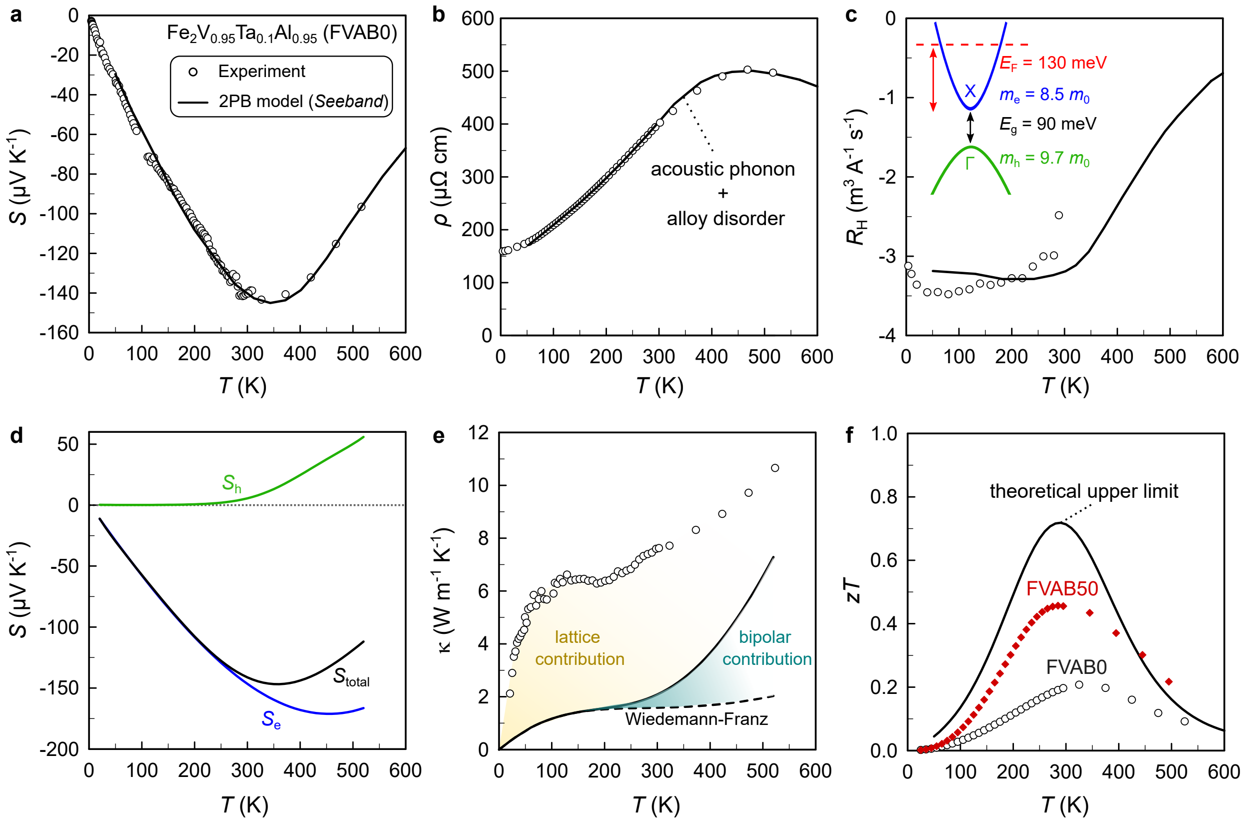

Fig. S14 | Modelling temperature-dependent electronic transport in Heusler-type $Fe_2V_{0.95}Ta_{0.1}Al_{0.95}$. **a**, Temperature-dependent Seebeck coefficient **b**, electrical resistivity and **c**, Hall coefficient. Solid lines are least-squares fits employing a two-parabolic band model, as implemented in the *SeeBand* fitting algorithm[3]. Two different scattering mechanisms have been considered: (i) Charge carrier scattering off acoustic phonons and (ii) alloy disorder scattering arising from Ta substitution and off-stoichiometry. The inset in **c** shows a sketch of the band structure. **d**, Total and individual band contributions to the temperature-dependent Seebeck coefficient. **e**, Temperature-dependent thermal conductivity. Solid line represents the total electronic part including the bipolar contribution, which can be calculated when $S(T)$, $\rho(T)$ and $R_{\mathrm{H}}(T)$ are approriately described within the electronic band structure model. Dashed line depicts the Wiedemann-Franz term of the electronic part, which significantly deviates from the total electronic contribution at high temperatures. **f**, Temperature-dependent zT from experiments and theoretical curve predicted by the two-band model in the limit that $\kappa_{\mathrm{L}} \to 0$ (solid line). Experimental data of FVAB50 composite are also added for comparison.

8.15. Temperature-dependent Seebeck modelling of $Bi_{0.9}Sb_{0.1}$ and $Fe_2V_{0.95}Ta_{0.1}Al_{0.95}$ starting materials

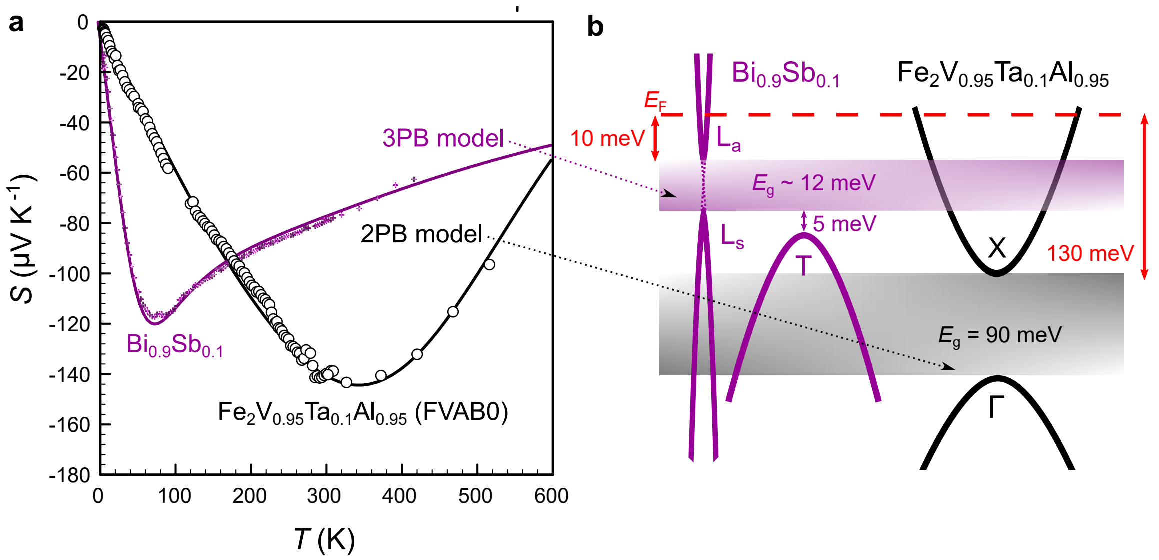

Fig. S15 | Three- and two-parabolic band modelling of the temperature-dependent Seebeck coefficient a, Temperature-dependent Seebeck coefficient of Heusler-type $Fe_2V_{0.95}Ta_{0.1}Al_{0.95}$ (FVAB0) and $Bi_{0.9}Sb_{0.1}$. Solid lines are least-squares fits employing a two- and three-parabolic band model, respectively. **b**, Sketch of the electronic band structure obtained from fitting $S(T)$. Energy gaps are displayed in milli electron volts and the position of the Fermi energy with respect to the band edges is displayed as a dashed red line.

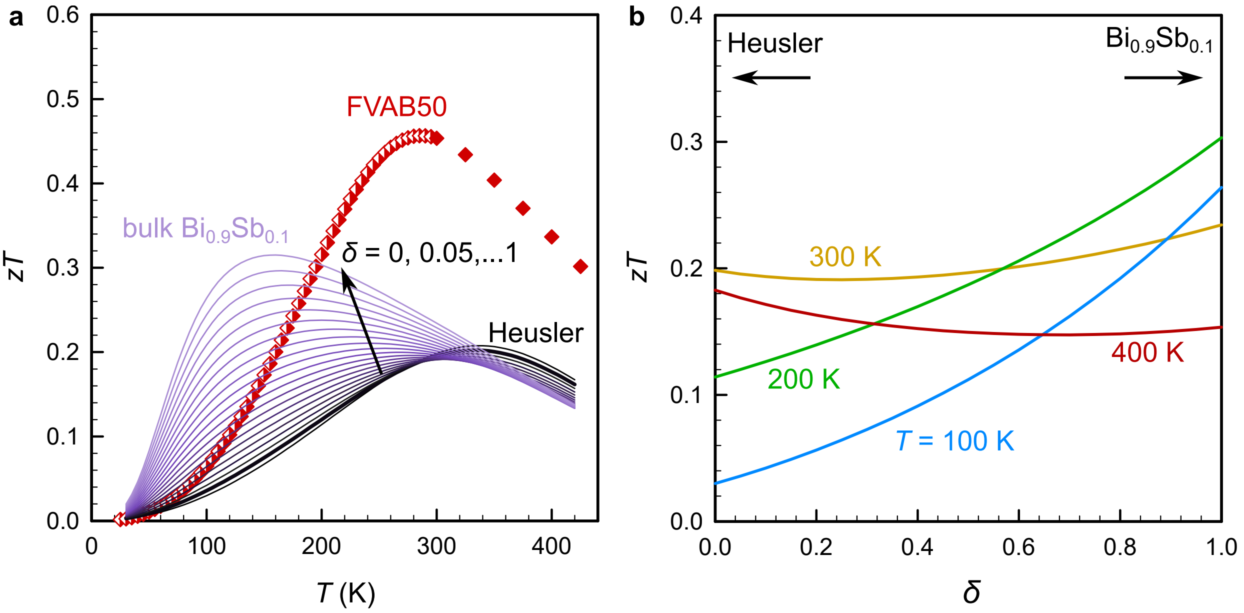

Fig. S16 | Volume fraction dependence of zT in effective medium theory. **a**, Temperature- and **b**, volume fraction-dependent zT of an effective medium comprising $Bi_{0.9}Sb_{0.1}$ in $Fe_2V_{0.95}Ta_{0.1}Al_{0.95}$ in a *parallel slabs* geometry (different geometries, e.g. random distribution, were calculated and yielded only minor differences for experimentally observed $\delta \approx 0.06$). In the framework of effective medium (EM) theory, the maximum figure of merit at any given temperature cannot exceed the maximum figure of merit of either component at the same temperature, irrespective of the geometry of the separate phases. Experimental data of the FVAB50 composite studied in this work, however, reveal a strong enhancement over EM theory, emphasizing a beneficial interplay between the two phases in the composite and/or a beneficial modification of the thermoelectric properties of the individual components.

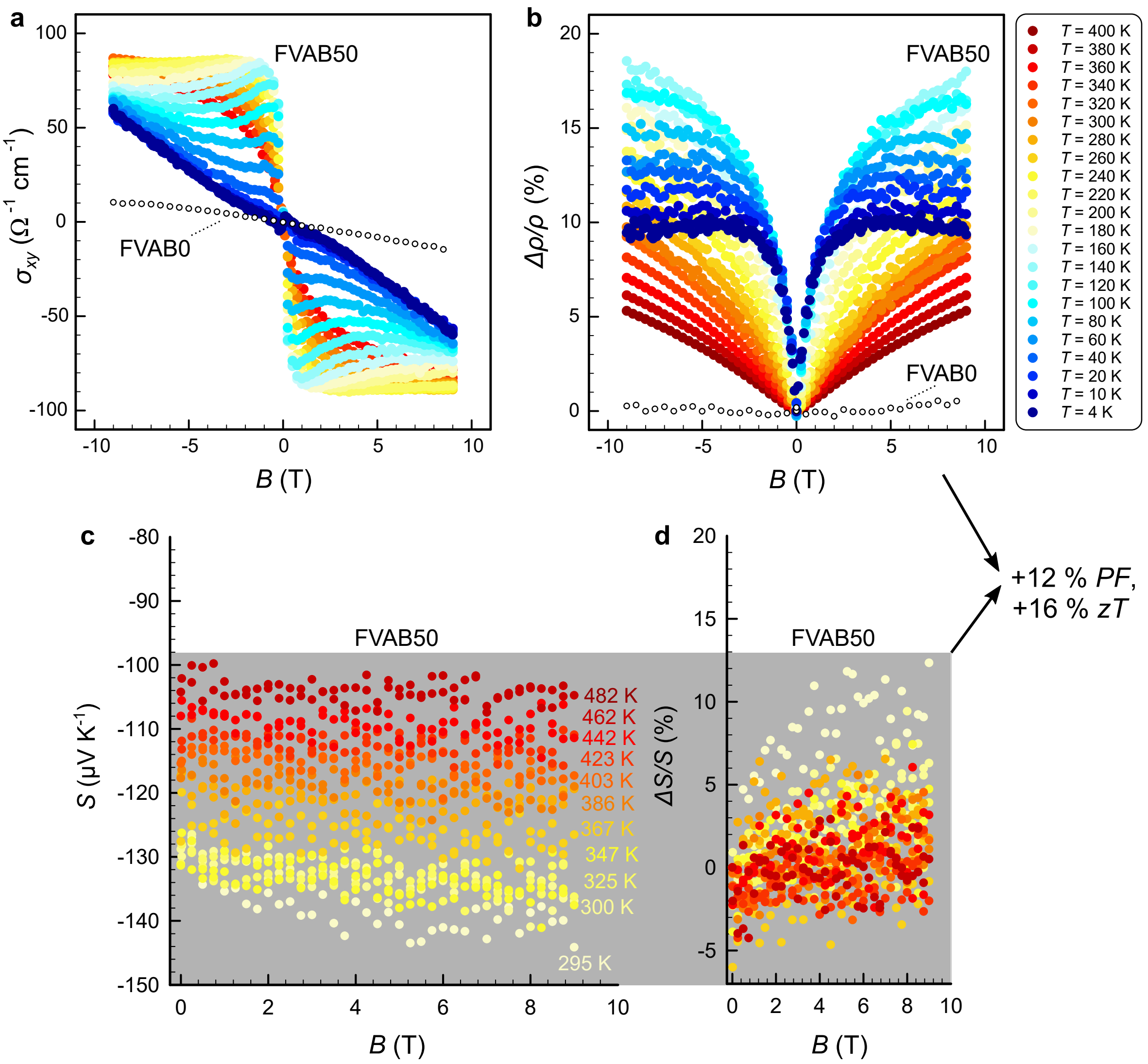

Fig. S17 | Additional field-dependent magneto-transport studies of FVAB50. **a**, Field-dependent, isothermal Hall conductivity $\sigma_{xy} = \rho_{xy}/(\rho_{xy}^2 + \rho_{xx}^2)$ and **b**, transverse magneto-resistance of FVAB50 from 2 to 400 K. Data for FVAB0 at 300 K are also shown for comparison. **c**,**d**, Field-dependent magneto-Seebeck effect of FVAB50 composite from 295 to 482 K.

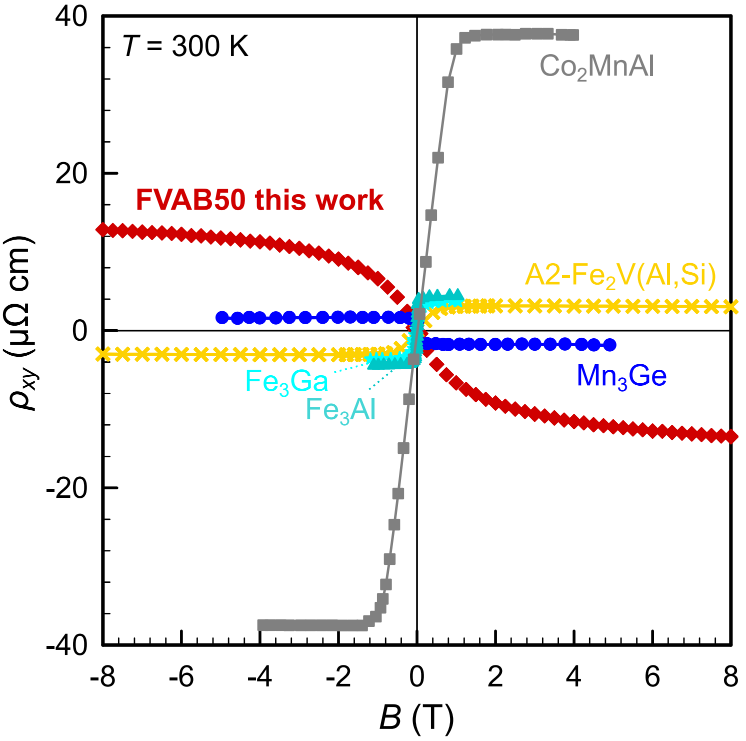

Fig. S18 | Comparison of anomalous Hall effect. Room-temperature anomalous Hall resistivity of nonmagnetic FVAB50 composite compared to other famous magnetic compounds with high anomalous Hall resistivities. The anomalous Hall effect in the former is attributed to a two-carrier channel transport of less mobile Heusler bulk carriers and Dirac-like surface electrons associated with the topological-insulating $Bi_{1-x}Sb_x$ secondary phase.

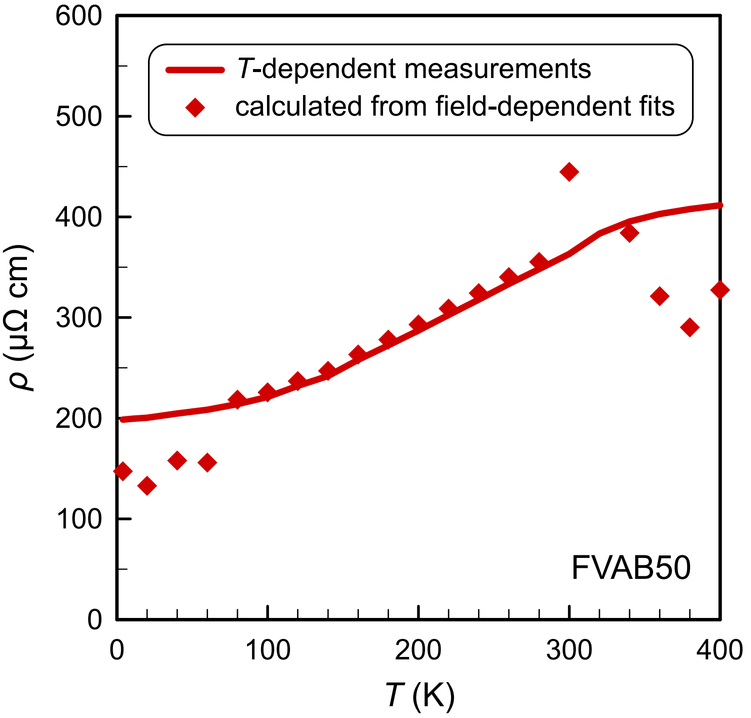

Fig. S19 | Robustness test of field-dependent Hall effect analysis. Temperature-dependent electrical resistivity of FVAB50 composite obtained directly from temperature-dependent measurements of $\rho_{xx}(T)$ and by recalculating $\rho_{xx}(T)$ from the Hall carrier mobilities and carrier concentrations derived from fitting $\rho_{xy}(B)$. The very good agreement between the two different methods highlights the robustness of the field-dependent Hall resistivity fits.

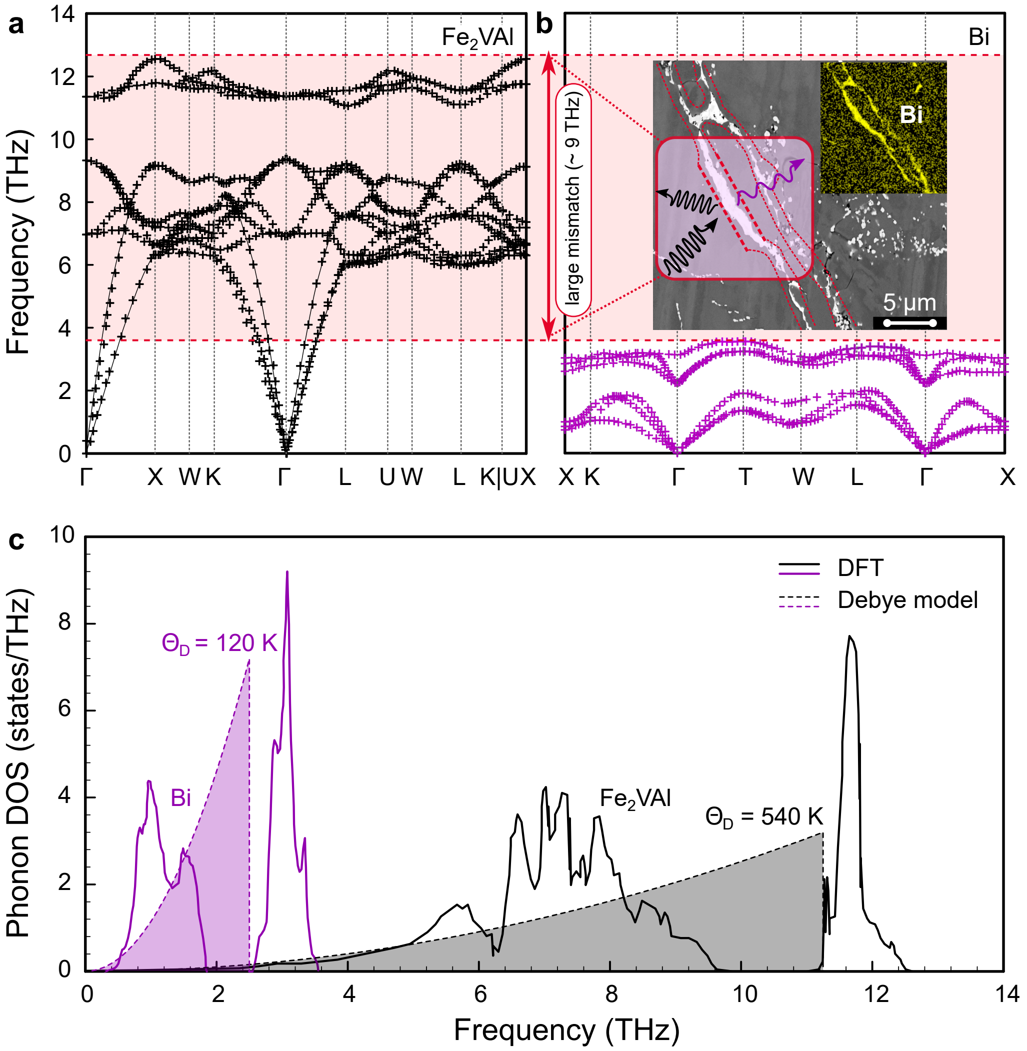

Fig. S20 | Phonon dispersions and densities of states. a,**b**, Comparison of the phonon band structure of Fe_2VAl and elemental Bi. The large mismatch of about 9 Thz in the phonon dispersion implies a strong phonon energy filtering effect (see inset). **c**, Phonon densities of states from density functional theory (DFT) and in the simple Debye model approximation. The large difference in the phonon spectra is reflected also in a substantial difference of the Debye temperatures.

8.21. Comparison of thermal conductivity in different directions of the sample

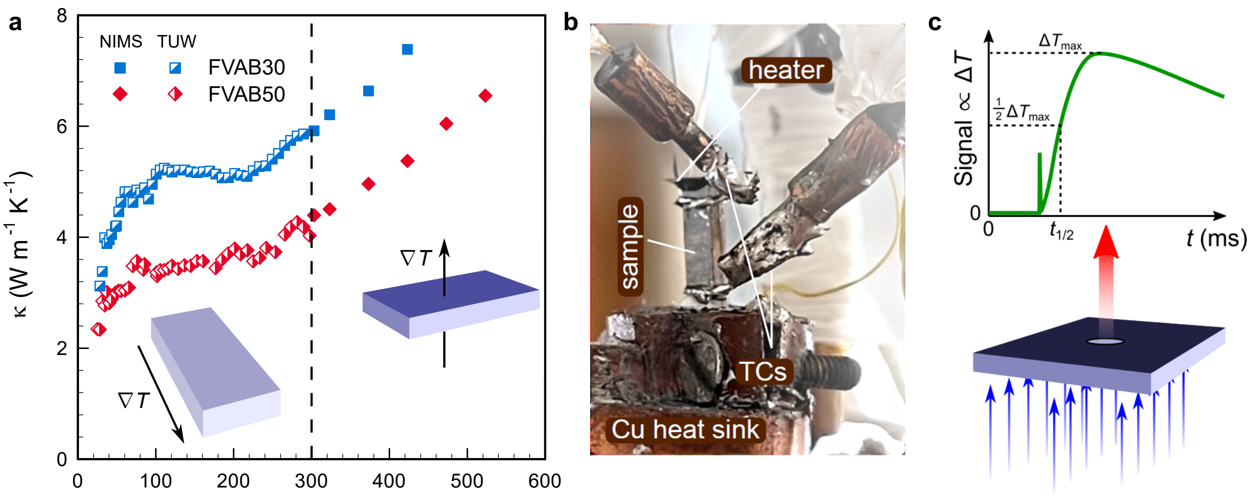

Fig. S21 | Isotropy of thermal transport in ($Fe_2V_{0.95}Ta_{0.1}Al_{0.95}$+$Bi_{0.9}Sb_{0.1}$) composites. **a**, Excellent agreement between low- and high-temperature measurements of the thermal conductivity rule out any anisotropy in the transport properties of the FVAB composite samples studied in this work. Low-temperature measurements were performed in the longitudinal direction, perpendicular to the compaction direction, using a steady-state method (**b**) employed in a home-built setup at TU Wien. High-temperature measurements were performed parallel to the compaction direction (see sketch in **c**), using a laser-flash method for the diffusivity and a differential scanning calorimeter for the specific heat, in a commercial setup (LFA 467, NETZSCH) at NIMS.

8.22. *Thermal stability of thermoelectric properties of FVAB50 for multiple measurement cycles*

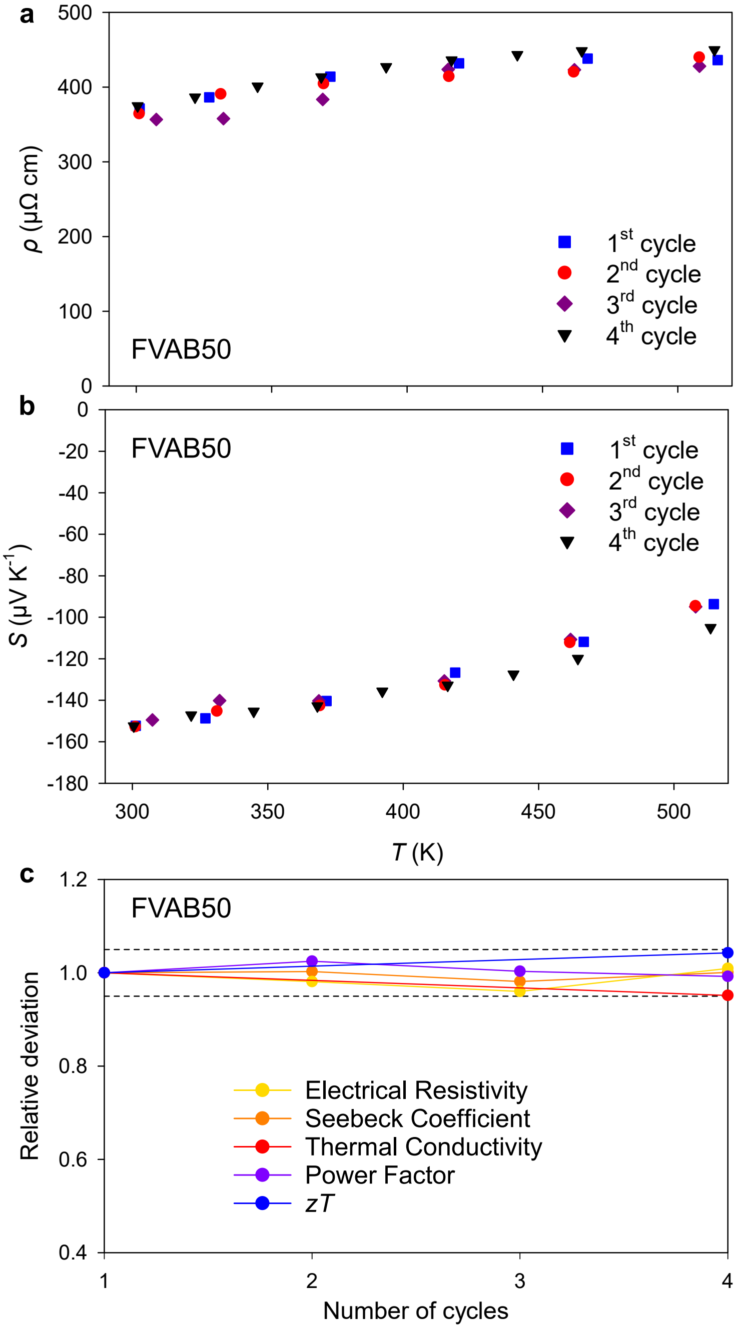

Fig. S22 | Thermal stability of FVAB50 composite. a, Temperature-dependent electrical resistivity and **b**, Seebeck coefficient of the high-performance FVAB50 composite for multiple thermal cycles. **c**, Relative deviation of electrical resistivity, Seebeck coefficient, thermal conductivity, power factor and zT versus the number of measurement cycles.

8.23. Temperature-dependent weighted mobility of FVAB50 composite versus many other n-type thermoelectric reported in the StarryData2 data base

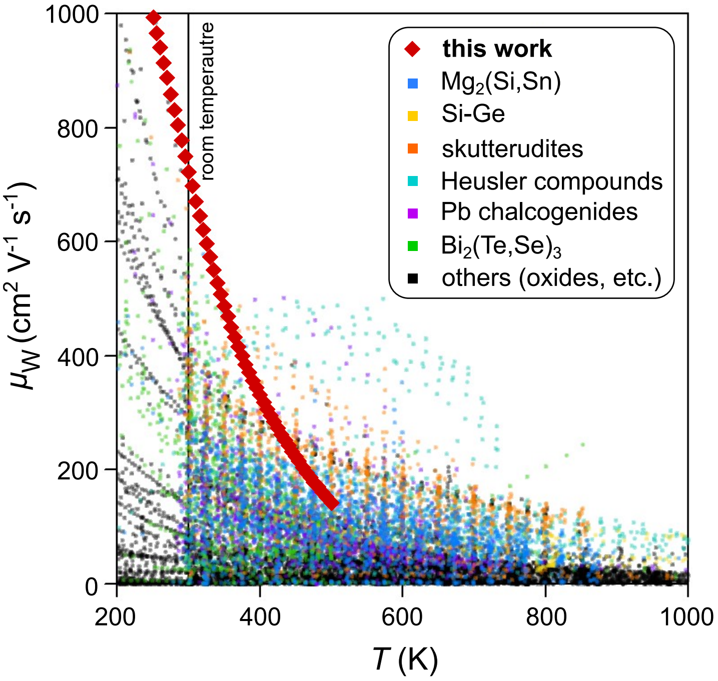

Fig. S23 | Comparison of weighted mobilities. Temperature-dependent weighted mobility of $Fe_2V_{0.95}Ta_{0.1}Al_{0.95}$ composite with 50 vol.% $Bi_{0.9}Sb_{0.1}$ added before sintering (FVAB50) compared to all other *n*-type thermoelectric semiconductors reported (as of July 2024) in the *Starrydata2* open web database[25].

Supplementary References

[1] Ashcroft, N. W. & Mermin, N. D. Solid state physics 1st ed. (1976).

[2] Eguchi, G. & Paschen, S. Robust scheme for magnetotransport analysis in topological insulators. *Physical Review B* **99**, 165128 (2019).

[3] Parzer, M. *et al.* SeeBand: A highly efficient, interactive tool for analyzing electronic transport data. *arXiv preprint arXiv:2409.06261* (2024).

[4] Putley, E. Galvano-and thermo-magnetic coefficients for a multi-band conductor. *Journal of Physics C: Solid State Physics* **8**, 1837 (1975).

[5] May, A. F. & Snyder, G. J. Introduction to modeling thermoelectric transport at high temperatures. *Materials, preparation, and characterization in thermoelectrics* 1–18 (2012).

[6] Masuda, S., Tsuchiya, K., Qiang, J., Miyazaki, H. & Nishino, Y. Effect of high-pressure torsion on the microstructure and thermoelectric properties of Fe_2VAl-based compounds. *Journal of Applied Physics* **124**, 035106 (2018).

[7] Rogl, G. *et al.* High-pressure torsion, a new processing route for thermoelectrics of high ZTs by means of severe plastic deformation. *Acta materialia* **60**, 2146–2157 (2012).

[8] Rogl, G. *et al.* Influence of shear strain on HPT-processed n-type skutterudites yielding ZT= 2.1. *Journal of Alloys and Compounds* **855**, 157409 (2021).

[9] Rogl, G. & Rogl, P. F. High pressure torsion, a large-scale manufacturing tool for high ZT skutterudite thermoelectrics. *Zeitschrift für anorganische und allgemeine Chemie* **648**, e202200044 (2022).

[10] Rogl, G., Zehetbauer, M. J. & Rogl, P. F. The effect of severe plastic deformation on thermoelectric performance of skutterudites, half-Heuslers and Bi-tellurides. *Materials Transactions* **60**, 2071–2085 (2019).

[11] Rogl, G. *et al.* Half-Heusler alloys: Enhancement of ZT after severe plastic deformation (ultra-low thermal conductivity). *Acta Materialia* **183**, 285–300 (2020).

[12] Fukuta, K., Tsuchiya, K., Miyazaki, H. & Nishino, Y. Improving thermoelectric performance of Fe_2VAl-based Heusler compounds via high-pressure torsion. *Applied Physics A* **128**, 184 (2022).

[13] Garmroudi, F. *et al.* Boosting the thermoelectric performance of Fe_2VAl-type Heusler compounds by band engineering. *Physical Review B* **103**, 085202 (2021).

[14] Hinterleitner, B. *et al.* Stoichiometric and off-stoichiometric full Heusler $Fe_2V_{1-x}W_xAl$ thermoelectric systems. *Physical Review B* **102**, 075117 (2020).

[15] Singh, S., Valencia-Jaime, I., Pavlic, O. & Romero, A. H. Elastic, mechanical, and thermodynamic properties of Bi-Sb binaries: Effect of spin-orbit coupling. *Physical Review B* **97**, 054108 (2018).

[16] Kawaharada, Y., Kurosaki, K. & Yamanaka, S. High temperature thermoelectric properties of $(Fe_{1-x}V_x)_3Al$ Heusler type compounds. *Journal of alloys and compounds* **349**, 37–40 (2003).

[17] Li, M. *et al.* Ultra-high thermoelectric performance in graphene incorporated Cu2Se: Role of mismatching phonon modes. *Nano Energy* **53**, 993–1002 (2018).

[18] Kim, C. & Lopez, D. H. Energy filtering and phonon scattering effects in Bi_2Te_3--PEDOT composite resulting in enhanced n-type thermoelectric performance. *Applied Physics Letters* **120** (2022).

[19] Kumar, A. & Wojciechowski, K. T. Effect of interface thermal resistance on thermoelectric properties of acoustically mismatched composite. *Journal of the European Ceramic Society* **42**, 4227–4232 (2022).

[20] Kumar, A. *et al.* Synergistic effect of work function and acoustic impedance mismatch for improved thermoelectric performance in GeTe-WC composite. *ACS Applied Materials & Interfaces* **14**, 44527–44538 (2022).

[21] Liang, Z. *et al.* Near-Room-Temperature Thermoelectric Performance Enhancement Via Phonon Spectra Mismatch in $Mg_3(Sb, Bi)_2$-Based Material by Incorporating Multi-Walled Carbon Nanotubes. *Advanced Energy Materials* **13**, 2301107 (2023).

[22] Kim, S. I. *et al.* Dense dislocation arrays embedded in grain boundaries for high-performance bulk thermoelectrics. *Science* **348**, 109–114 (2015).

[23] Deng, R. *et al.* Thermal conductivity in $Bi_{0.5}Sb_{1.5}Te_{3+x}$ and the role of dense dislocation arrays at grain boundaries. *Science Advances* **4**, eaar5606 (2018).

[24] Heremans, J. Final Project Report: Cryogenic Peltier Cooling. *The Ohio State University* **AFRL-AFOSR-VA-TR-2017-0084**.

[25] Katsura, Y. *et al.* Data-driven analysis of electron relaxation times in PbTe-type thermoelectric materials. *Science and Technology of Advanced Materials* **20**, 511–520 (2019).
